# Short-term expansion of breast circulating cancer cells predicts response to anti-cancer therapy

**DOI:** 10.18632/oncotarget.3903

**Published:** 2015-05-06

**Authors:** Bee Luan Khoo, Soo Chin Lee, Prashant Kumar, Tuan Zea Tan, Majid Ebrahimi Warkiani, Samuel GW. Ow, Sayantani Nandi, Chwee Teck Lim, Jean Paul Thiery

**Affiliations:** ^1^ Mechanobiology Institute, National University of Singapore, Singapore; ^2^ Department of Hematology-Oncology, National University Cancer Institute, National University Hospital, Singapore; ^3^ Cancer Science Institute of Singapore, National University of Singapore, Singapore; ^4^ Institute of Molecular and Cell Biology, A*STAR (Agency for Science, Technology and Research), Singapore; ^5^ BioSystems and Micromechanics (BioSyM) IRG, Singapore-MIT Alliance for Research and Technology (SMART) Centre, Singapore; ^6^ School of Mechanical and Manufacturing Engineering, University of New South Wales, Sydney, Australia; ^7^ Department of Biomedical Engineering, National University of Singapore, Singapore; ^8^ Department of Mechanical Engineering, National University of Singapore, Singapore; ^9^ Department of Biochemistry Yong Loo Lin School of Medicine, National University of Singapore, Singapore

**Keywords:** breast cancer, circulating tumor cells, CTCs, enrichment

## Abstract

Circulating tumor cells (CTCs) are considered as surrogate markers for prognosticating and evaluating patient treatment responses. Here, 226 blood samples from 92 patients with breast cancer, including patients with newly diagnosed or metastatic refractory cancer, and 16 blood samples from healthy subjects were cultured in laser-ablated microwells. Clusters containing an increasing number of cytokeratin-positive (CK+) cells appeared after 2 weeks, while most blood cells disappeared with time. Cultures were heterogeneous and exhibited two distinct sub-populations of cells: ‘Small’ (≤ 25 μm; high nuclear/cytoplasmic ratio; CD45-) cells, comprising CTCs, and ‘Large’ (> 25 μm; low nuclear/cytoplasmic ratio; CD68+ or CD56+) cells, corresponding to macrophage and natural killer-like cells. The Small cell fraction also showed copy number increases in six target genes (FGFR1, Myc, CCND1, HER2, TOP2A and ZNF217) associated with breast cancer. These expanded CTCs exhibited different proportions of epithelial–mesenchymal phenotypes and were transferable for further expansion as spheroids in serum-free suspension or 3D cultures. Cluster formation was affected by the presence and duration of systemic therapy, and its persistence may reflect therapeutic resistance. This novel and advanced method estimates CTC clonal heterogeneity and can predict, within a relatively short time frame, patient responses to therapy.

## INTRODUCTION

Circulating tumor cells (CTCs), derived from either primary or metastatic tumors, have raised considerable interest within the translational oncology community [1, 2]. CTCs appear during the early stages of tumor progression [3, 4] as single cells or cell clusters and exhibit a partial or complete epithelial–mesenchymal transitioned (‘EMTed’) phenotype [5, 6]. These cells may later colonize distant organs and develop into clinically detectable metastases. Thus, studies have proposed that CTCs could be used to provide an assessment of the current tumor status via “liquid biopsy” [7–9] or may serve as surrogate markers for patient responses to treatment [2, 10] and offer some guidance as to the choice of therapeutic intervention.

Nonetheless, CTCs are rare events, and must be enriched prior to genomic and proteomic analyses. Conventional assays detect only low numbers of CTCs and this poses a significant challenge for defining their characteristics [10, 11], particularly since they do not express a targetable marker. Consequently, there is an urgent need for techniques that can successfully expand CTCs. Recent studies have reported the establishment of cell lines derived from CTCs of breast [12, 13], colon [14] and prostate [15] cancer patients, obtained following pre-enrichment with affinity binding [12], fluorescence-activated cell sorting (FACS) [13], or negative selection [15]. However, the efficiencies in obtaining CTC cultures using these methods were low (< 20%), and the need for pre-enrichment with each method resulted in a loss of CTC count. Hence, there is still a need for a method with improved efficiency and without the need for prior enrichment for practical use in the clinic.

Non-adhesive substrates are typically recommended for culturing cancer stem-like cells from primary tumors, and these cultures tend to generate multilayered cell clusters [16, 17]. Cluster formation can also be promoted using microwells, as described elsewhere for embryonic stem cells [18, 19] or cancer cell lines [20]. In addition, it has been indicated that hypoxic conditions (1% O_2_) can promote cellular reprogramming towards a cancer stem cell (CSC) phenotype [21, 22]. In consideration of these previous results, we designed and explored the potential clinical utility of a novel culture scheme using laser-ablated microwells that permits the expansion of CTCs from the whole blood of patients with early stage, locally advanced, or metastatic breast cancers. We show that, within the nucleated blood cell fraction, most blood cells (leukocytes, mesenchymal stem cells [23]) and endothelial cells are progressively eliminated from the culture over time, allowing for the selective enrichment of CTCs, which go on to form proliferative clusters. We also demonstrate that these cultured cells comprise two distinct sub-populations: the smaller-sized (≤ 25 μm), CD45-negative CTC fraction, with a high nuclear/cytoplasmic (N/C) ratio, and the larger (> 25 μm) CD68-positive monocytes/macrophages or CD56-positive natural killer [24]-like cells, with a low N/C ratio. In further assessment of the small cell fraction, we show that these cells express either or both epithelial and mesenchymal markers as well as copy number increases in six genes previously identified to be altered in breast cancers. Furthermore, cluster formation in cultures is significantly reduced with samples obtained from patients who had undergone at least one treatment cycle as compared with those samples from untreated patients. Therefore, we surmise that cluster formation may provide a unique and predictive method for treatment response and efficacy.

## RESULTS

### CTC culture, characterization and cluster formation

**Figure 1:**
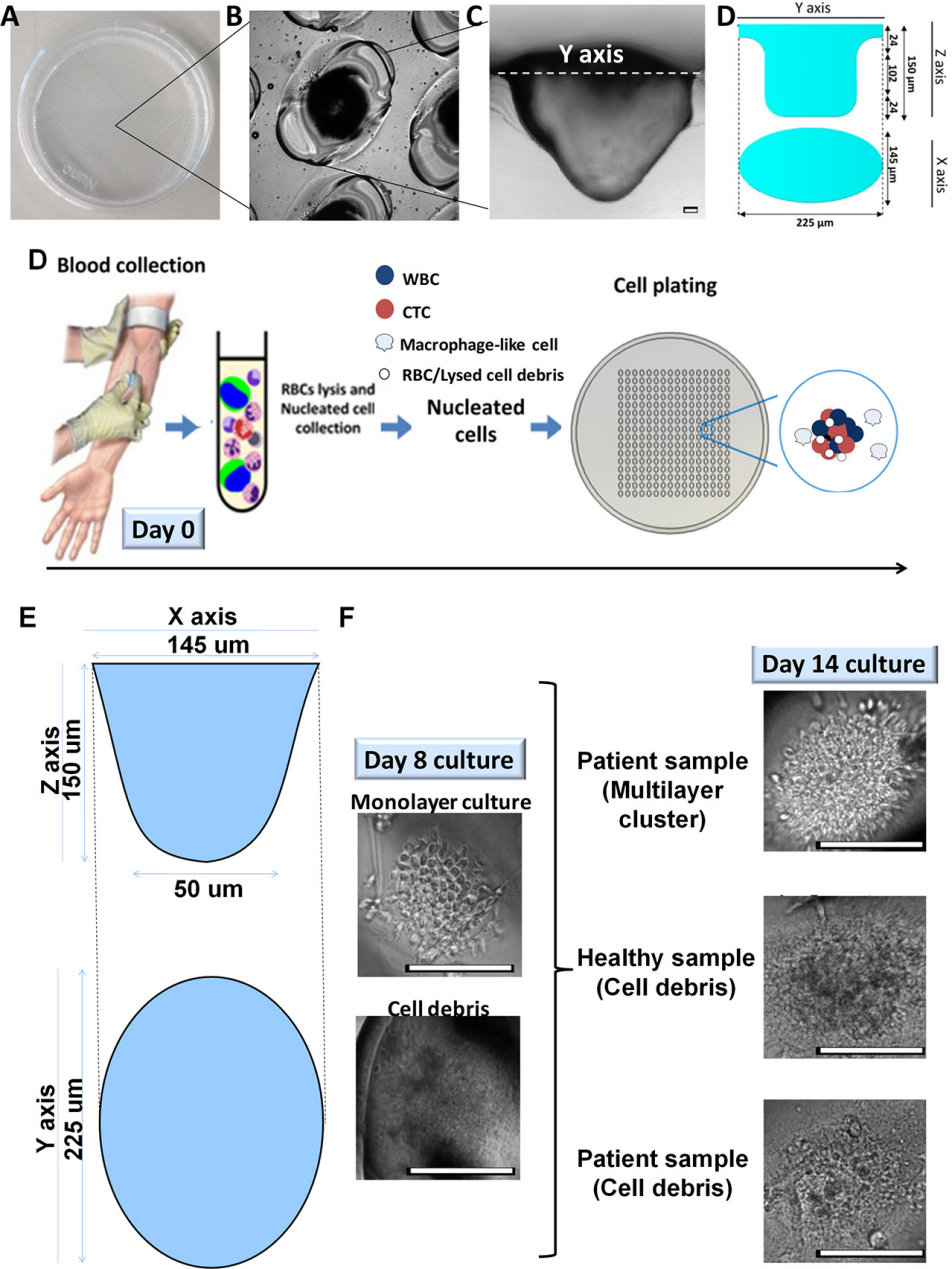
Overview of microwell-based culture technique for CTC expansion **A.** Microwell assay is represented by an image of an actual patterned dish with laser-ablated microwells **B.** Close-up of the microwells at 20 × magnification. **C.** Cross-section of a polydimethylsiloxane (PDMS) replica for one of the microwells. Scale bar, 10 μm. **D.** Preparation of the nucleated cell fraction from peripheral blood via red blood cell (RBC) lysis. **E.** A schematic diagram displaying estimated dimensions of a microwell. The ellipsoidal tapered microwell has major and minor diameters of 225 μm and 145 μm, respectively, at the opening of the well, and a depth of 150 μm. **F.** At Day 8, cultures may appear as a monolayer of cells within microwells, while some of the non-proliferative cultures may have already generated cell debris. Proliferative cultures (top right, patient sample) proceed to form multilayered clusters by Day 14, whereas non-proliferative cultures (second and bottom right, healthy and patient samples) generate cell debris. Scale bar, 100 μm.

A laser-ablated, microwell-based assay (Figure 1A–1D) for CTC culture was established with nucleated cells from patient blood samples (Supplementary Tables 1A–1D) after red blood cell (RBC) lysis (Figure 1D). We sought to culture these cells for up to 14 days. At Day 8, most cultures appeared as a monolayer of cells (Figure 1F, left). By Day 14, the proliferative cultures proceeded to form multilayered clusters, whereas the non-proliferative cultures generated a noticeable amount of cell debris (Figure 1F, right bottom). The clusters formed in the proliferative cultures varied in diameter (Supplementary Figure 2B) and predominantly consisted of a heterogeneous collection of non-senescent cells (~90.1% of β-galactosidase-negative cells; Supplementary Figure 3).

CTCs immunocaptured by EpCAM antibodies in devices [25, 26], including the FDA-approved CellSearch [27], are often identified as cytokeratin (CK)-positive, CD45-negative cells exhibiting a high N/C ratio. We therefore estimated the proportion of CTCs in our cultures in a similar manner, using immunostaining for CK and CD45 concomitant with a nuclear stain (Hoechst), and using N/C ratio determination. Day 14 proliferative cultures were harvested and analyzed, as summarized in the flow chart in Supplementary Figure 1. Cells were separated into two populations based on size using a spiral inertia microfluidic device [28]. The resultant subpopulations, hereafter referred to as ‘Small’ (≤ 25 μm) and ‘Large’ (> 25 μm) cells, were morphologically differentiated using *Papanicolaou* [29] and Diff-QUIK staining (Supplementary Figure 2A). The Large cells were well differentiated and had a low N/C ratio, whereas the Small cells exhibited strongly stained nuclei and a high N/C ratio, features of a malignant phenotype. These cultures also showed variable CK expression, with CK+ cells localized in the center of the well, surrounded by CD45+ cells (Supplementary Figure 4A). A significant number of these CK+ cells also expressed vimentin (Supplementary Figure 4B), suggesting a transition of these cells from an epithelial to an intermediate EMT phenotype. Most of the large cells within and outside the microwells expressed CD68, which is suggestive of macrophages (Supplementary Figure 4C; Supplementary Methods). The macrophage-like behavior of these cells was confirmed with 1-μm fluorescein-labeled polystyrene microbeads that were phagocytosed within a 24-h time frame (Supplementary Figure 4D). Outside the microwells, we detected some detached cell clumps, consisting of small cells only, and these cells were negative for CD68 (Supplementary Figure 4C).

**Figure 2:**
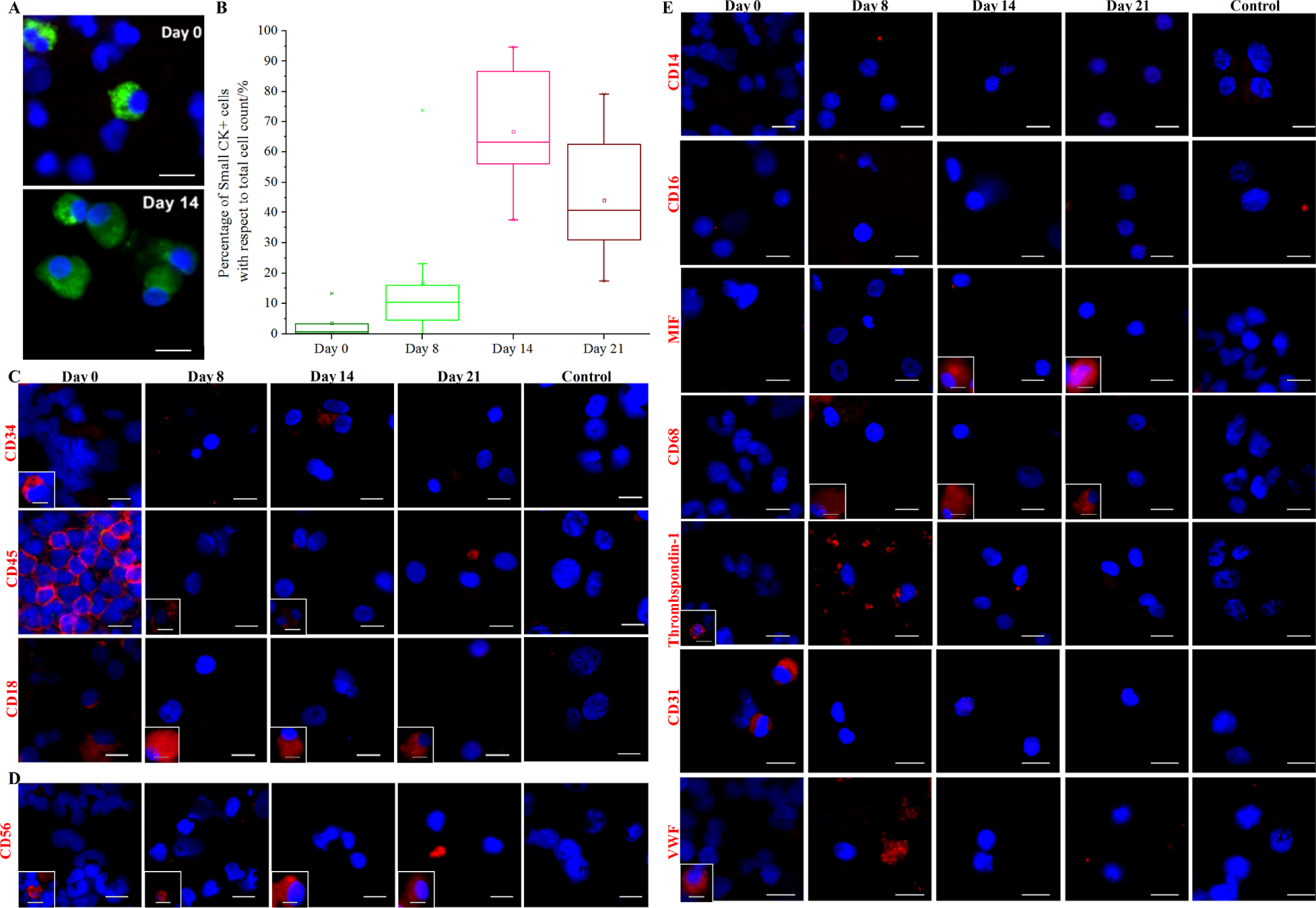
Expansion of CK+ cells and depletion of blood cells in culture **A.** Immunostaining (pan-CK-FITC, Hoechst) of cytospots obtained from culturing blood samples harvested at different time points (Days 0, 8, 14 and 21). Scale bar, 20 μm. **B.** Percentage of Small CK+ cells (15–25 μm) with respect to total cell count (Hoechst+) at various time points (Days 0, 8, 14 and 21). Significant expansion of CK+ cells can be observed by Day 14. **C.** Immunostaining of hematopoietic precursors and leukocytes. Boxed images (marked in white) provide examples of a distinct minority phenotype from the majority of cells. CD34+ cells (hematopoietic precursors) disappeared from culture with time. A minority of CD45+ and CD18+ cells persist in culture. Negative control (MDA-MB-231 cell line) for each antibody is provided (last column). Scale bar, 20 μm. **D.** Immunostaining for the natural killer cell marker, CD56. Minority populations of CD56+ (~22.2% ± 9%) persist in culture. Boxed images (marked in white) provide examples of a distinct minority phenotype from the majority of cells. Negative control (MDA-MB-231 cell line) (to determine antibody specificity) is provided in the last column. Scale bar, 20 μm. **E.** Immunostaining of specific white blood cell (WBC) and endothelial cell markers. Boxed images (marked in white) provide examples of a distinct minority phenotype from the majority of cells. Cultured cells are generally negative for thrombospondin-1, CD14, CD16, von Willebrand factor (VWF) and CD31. Minority populations of CD68+ and MIF+ (migration inhibitory factor) cells (~33% ± 26%) persist in culture. Negative control (MDA-MB-231 cell line) for each antibody is provided (last column). Scale bar, 20 μm.

We next sought to compare the proportions of CK+/CD45- Small cells in cultures at Days 0 (nucleated fraction), 8, 14 and 21 (Figure 2A; Supplementary Figure 5) using cytospot preparations of the cultures; the MDA-MB-231 cell line was used as a negative control. We found that the Small CK+ CTC counts increased over time with respect to total cell counts (Figure 2B), and that these increases correlated with the initial abundance of CK+ CTCs in the blood before culture; albeit, some blood samples that did not initially contain detectable CK+ CTCs were later positive at Day 14 (Supplementary Table 2). The proportion of CK+/CD45- cells decreased significantly after Day 14 for most samples (Figure 2B, Supplementary Figure 5); therefore, we selected Day 14 as the end-point for culture phenotyping. This time-point also correlated with the highest number of Ki67-positive clusters (Supplementary Figure 6).

Interestingly, we noted that the proportion of CTCs relative to the total cell count varied across the samples examined (*n* = 10), ranging from 37.5% to 94.6% (Figure 2B). Non-proliferative blood cells present in the Day 0 nucleated fraction resulted in cell debris that was progressively eliminated with media changes. Macrophages (~33% ± 26%) and NK cells (~22.2% ± 9%) were identified using leukocyte markers (CD45 and CD18; Figure 2C), a NK cell marker (CD56; Figure 2D), and macrophage markers (migration inhibitory factor, MIF, and CD68; Figure 2E). Blood cells of other lineages were rarely noted, as revealed by immunostaining for hematopoietic precursors (CD34; Figure 2C), monocytes (CD14 and CD16), megakaryocytes (thrombospondin-1) and endothelial cells (CD31 and von Willebrand factor; Figure 2D). Cells expressing mesenchymal stem cell (MSC)-associated markers were also rarely detected, as determined using antibodies against CD90 and various markers of differentiation (aggrecan for chondrocytes, FABP4 for adipocytes, osteocalcin for osteocytes, and troponin T for cardiomyocytes; Supplementary Figure 7). Overall, the data demonstrate that cultured cells from cancer patients consisted predominantly of CK+/CD45- CTCs, macrophages, and NK cells.

Finally, we compared these cultures with those of blood samples taken from 16 healthy subjects (Supplementary Table 3). Blood samples from healthy subjects generated monolayers with cell debris (Figure 1E, Supplementary Figure 8A). The cells from the monolayer were CK+/Hoechst+/CD68+, confirming that they were macrophages (Supplementary Figure 8B).

### Enrichment of phenotypes with cancer stem cell (CSC) markers

Tumor-initiating cells have been shown to carry stem cell-like properties [17, 30], as well as drug resistance [31, 32] and drug tolerance [33–35]. Breast cancer stem cells (CSCs), initially identified as CD44+/CD24- cells [17], are also known to express EMT markers [17, 36]. In several other studies, these CSCs have been detected as a subpopulation in CTC cultures [37–40]. Thus, we next sought to evaluate the presence of CD44+/CD24- cells in our culture, and determined the impact of hypoxia and a microwell-based culture system on the expansion of these putative CSCs. Cultures were maintained in one of three conditions, each performed in triplicate: hypoxia (10% serum, 1% oxygen) in microwells, hypoxia on 2D-uncoated substrates, or normoxia in microwells. Blood cells were present in all three conditions (Supplementary Figure 9A), but clusters only formed in the hypoxia/microwell condition after one week in culture. The proportion of CD44+/CD24- cells was lower on the 2D-uncoated substrates or under normoxic conditions, as compared with cultures maintained in microwells under hypoxic conditions (Supplementary Figure 9B). The estimated ratio of CD44+/CD24- cells was 1:0.62:0.42 (hypoxia/microwell: hypoxia/2D substrate: normoxia/microwell; Supplementary Figure 10). Day 14 cultures under hypoxia/microwell conditions were also positive for Rex1, an embryonic stem cell (ESC) marker (78.5% ± 17%; Supplementary Figure 11), but negative for other ESC markers, SOX2, Oct4 and Nanog. We also found subpopulations of cells with stem cell-like properties, as supported by spheroid formation in 3D Geltrex® or on ultra-low adhesive dishes at Day 14 (Supplementary Figure 12); this suggested the existence of tumorigenic cells. CD44+/CD24- cells were distinctly absent from cultures obtained from these healthy samples (Supplementary Figure 8C).

### Cultured CTCs are heterogeneous and contain mesenchymal-associated genes

**Figure 3:**
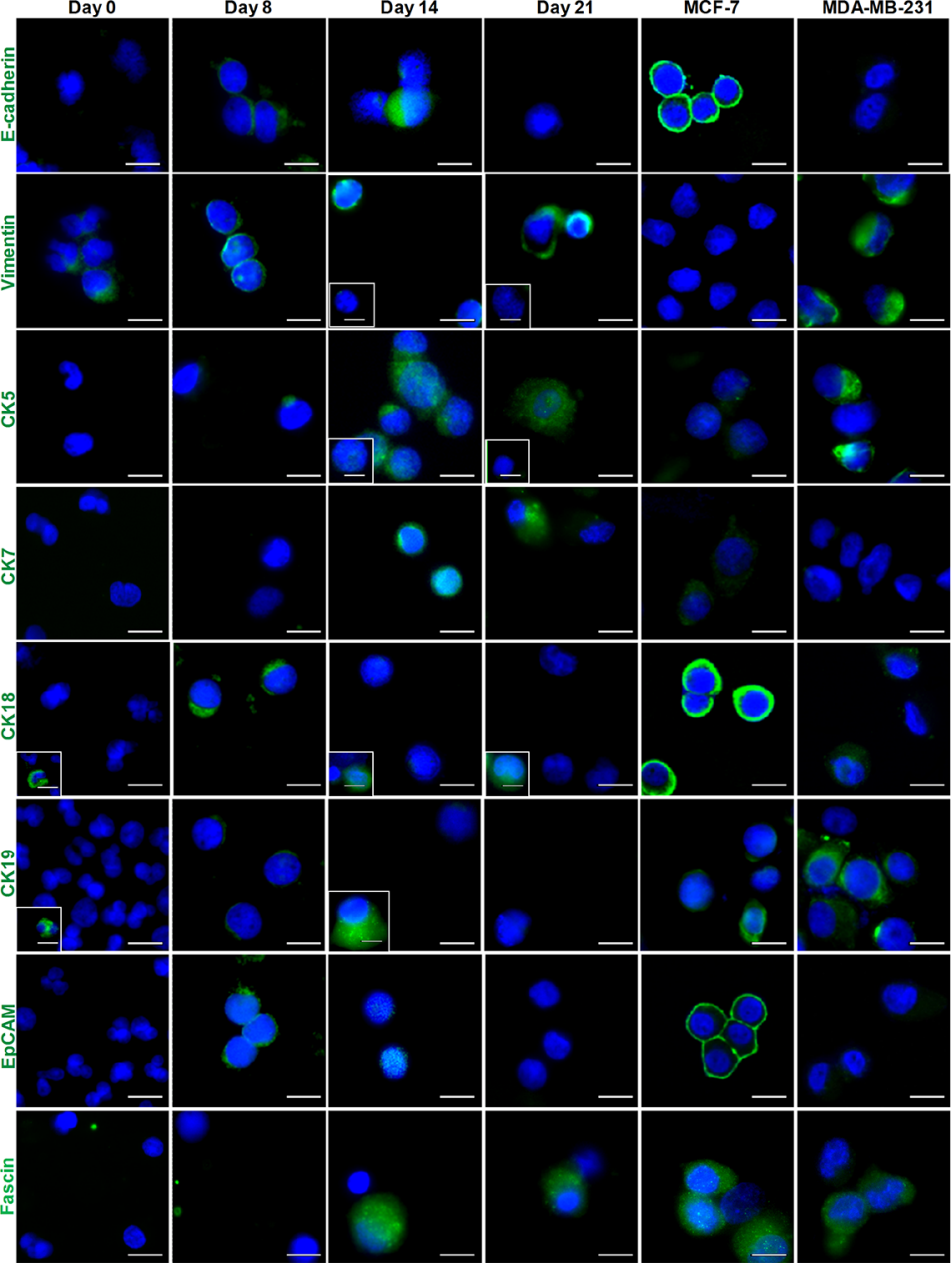
Immunostaining of epithelial and mesenchymal markers for Day 14 cultures Boxed images (marked in white) provide examples of a distinct minority phenotype from the majority of cells. Cells generally demonstrated increased expression of mesenchymal markers (Vimentin and Fascin), and decreased expression of epithelial markers (EpCAM and E-cadherin). Individual cytokeratin staining (CK5, CK7, CK18 and CK19) demonstrates that the cultured cells are more positive for CK5 and CK7 than CK18 and CK19. MCF-7 and MDA-MB-231 breast cancer cell lines were used as references for epithelial and mesenchymal carcinoma cell lines, respectively. Scale bar, 20 μm.

We next characterized the expression of epithelial and mesenchymal markers in the Small cell population using six epithelial markers (E-cadherin, CK5, CK7, CK18, CK19 and EpCAM) and two mesenchymal markers (Vimentin and Fascin) (Supplementary Table 4). MCF-7 and MDA-MB-231 cell lines were used as references for epithelial and mesenchymal carcinomas, respectively. Individual CK immunolabelling demonstrated that cultured cells express higher levels of CK5 and CK7 as compared with CK18 and CK19. Furthermore, cultured cells became increasingly more mesenchymal-like with time in culture, with increased Vimentin and Fascin staining and reduced or absent staining of epithelial markers (E-cadherin and EpCAM; Figure 3). The EMT status of CTCs at Day 14 was heterogeneous, with the majority of cells staining positively for both pan-CK and Vimentin antibodies (> 50%).

To better estimate the epithelial-like and mesenchymal-like sub-populations in these cultured CTCs, we used RNA FISH on 10 samples and assessed the expression of nine epithelial genes (CK7, CK8, CK18, CK19, CDH1, TFF1, FOXA1, AGR2 and GATA3) and four mesenchymal genes (PTX3, SERPINE2, VIM, FASCIN) (Supplementary Table 5, Supplementary Figure 13; Supplementary Methods). Cells were classified as Epithelial (E; mostly green fluorescence), Epithelial–Mesenchymal (EM; mixed fluorescence) or Mesenchymal (M; mostly red fluorescence), and MCF-7 and MDA-MB-231 cells were again used as phenotypic controls. The results showed that the phenotypes of Day 14 samples were indeed mixed, and this was irrespective of their estrogen receptor (ER), progesterone receptor (PR) or HER2 statuses.

### Copy number increase in breast cancer-associated genes

**Figure 4:**
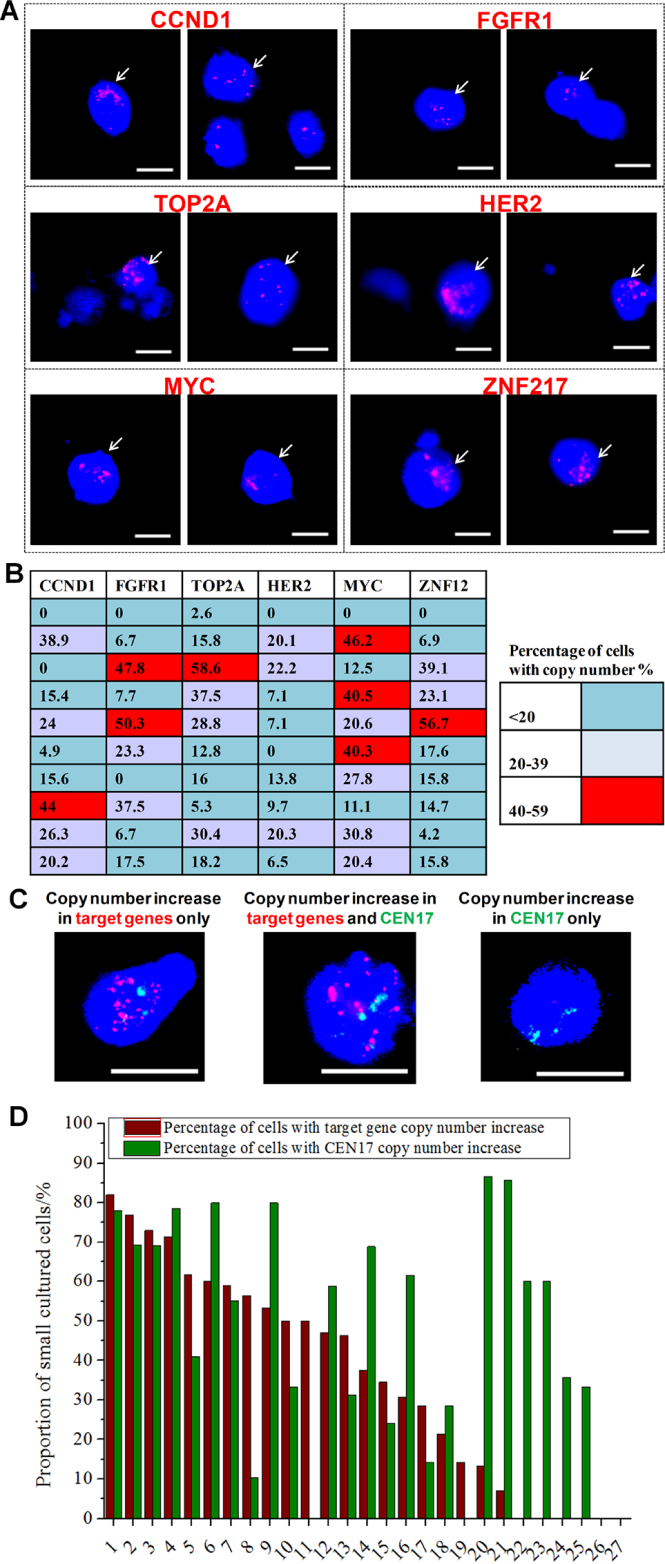
Genomic characterization of cultured CTCs **A.** Merged images (bright field, DAPI, spectrum green, spectrum orange) of DNA fluorescence *in situ* hybridization (FISH)-processed cultured cells processed separately with six target probes (FGFR1, MYC, CCND1, HER2, TOP2A and ZNF217, all red) corresponding to 50% of breast cancer types. Copy number increase in these genes can be observed in a proportion of the cultured cells (≥ 3 red signals per cell). Scale bar, 20 μm. **B.** Heat map representation of the proportion of cells in cultures (*n* = 10) with copy number increase in each of the six genes. 6/10 samples have 40% or more cells with copy number increase in 1 or more genes. **C.** Merged images (bright field, DAPI, spectrum green, spectrum orange) of DNA FISH-processed cultured cells using all six target probes (FGFR1, MYC, CCND1, HER2, TOP2A and ZNF217, all red) in each sample, demonstrating copy number increase for target genes in contrast to copy number of centromere for chromosome 17 (CEN17, green). Scale bar, 20 μm. **D.** Quantification for the proportion of ‘Small’ cells (15–25 μm) with target gene and/or CEN17 copy number increase in 27 cultured samples. Cells with copy number increase in target genes were determined as those which expressed ≥ 13 red signals. Cells with copy number increase in CEN17 were determined as those that expressed ≥ 3 green signals. Numerous samples (21/27) had a proportion of cells with target gene copy number increase, whereas almost all samples (25/27) had a proportion of cells with CEN17 copy number increase. The prevalence of the six target gene copy number increase is detected in ~44% of all breast cancers. Each bar corresponds to the respective sample as numbered (*x*-axis).

Six genes have been reported to contribute to about 44% of driver mutations in breast cancer (copy number increase or amplicons): MYC, FGFR1 (Chromosome 8); CCND1 (Chromosome 11); HER2, TOP2A (Chromosome 17); and ZNF217 (Chromosome 20) [24, 41]. We next employed DNA FISH to evaluate the amplification status of these six genes in Day 14 cultured cell samples (Supplementary Table 5; Supplementary Methods). First, we used single probes to ascertain the cells with copy number increase for each of the six genes (Figure 4A), with an increase defined as those cells with three or more red signals. In the 10 samples tested, all (10/10, 100%) showed a gain in at least one investigated gene locus. However, only 6/10 samples (60%) showed 40% or more cells with copy number increases in 1 or more genes (Figure 4B).

Next, we compared the total proportion of cells with a copy number increase in any of these probes with a concomitant increase in CEN17 copy number (an indicator of cell polyploidy and cancer progression). This was performed using another 27 samples, with all six probes used for each sample (Figure 4C). For this assay, the threshold for signals was increased to ≥ 13 red signals to indicate copy number increases in the target probe(s); cells with ≥ 3 green signals were considered to have copy number increase in CEN17. We found that cultured cells with single or multiple CEN17 signals had a copy number increase in one or more target probes, with 21/27 (77.8%) samples showing a proportion of cells with target gene copy number increase (range, 7.1%–80%; mean, 35.9%) and 25/27 (92.6%) samples showing a proportion of cells with a copy number increase in CEN17 (range, 10.3%–85.7%; mean, 46.2%; Figure 4D). There was no distinct correlation between CEN17 polysomy and target gene amplification in cultured CTCs, which is similar to that reported in other studies [42]. Overall, the detection of copy number increases in cancer-associated genes within the Small cultured cell population confirms the presence of cancer cells, and we surmise that these Small cells were likely derived from CTCs.

### Cultures predict response to anti-cancer therapy

Given that CTCs are considered to be surrogate markers for prognosticating and evaluating patient treatment responses, we next sought to determine the utility of the CTC cluster formation assay as a predictor of treatment response. To this end, we analyzed 173 pre- and/or post-treatment blood samples from 60 patients with early stage or metastatic breast cancer, who were receiving anti-cancer therapy and who had clinically measurable tumors. These patients had been enrolled into one of three clinical studies—two neoadjuvant trials (Cohort P2A/2B, Cohort PCL) and one metastatic study (Cohort CTB) (Supplementary Tables 1A–1D)—and blood samples were taken from each patient before treatment and at various time points during their treatment course and used for analysis. All patients gave their informed consent. Overall, we found that cluster formation (Figure 1) was seen progressively less frequently in blood samples from patients who had undergone longer durations of systemic therapy (pre-treatment: 39/44 (88.6%); < 3 weeks post-treatment: 27/32 (84.4%); 3–5 weeks post-treatment: 24/36 (66.7%) > 5 weeks post-treatment: 26/61 (42.6%), *p* < 0.001).

We thus next aimed to link clinicopathological factors with cluster formation in these cultures. Of the 5/44 (11.3%) pre-treatment samples that did not form clusters, two were from patients with invasive lobular carcinoma (ILC). Of the 5/32 (15.6%) < 3 weeks post-treatment samples that did not form clusters, one was from a patient with ILC whose baseline sample did not form clusters, whereas another was from a patient who went on to achieve pathological complete response after 12 weeks of neoadjuvant chemotherapy. These findings suggest the potential for cultured CTCs to be used as an early predictor of treatment response.

**Figure 5:**
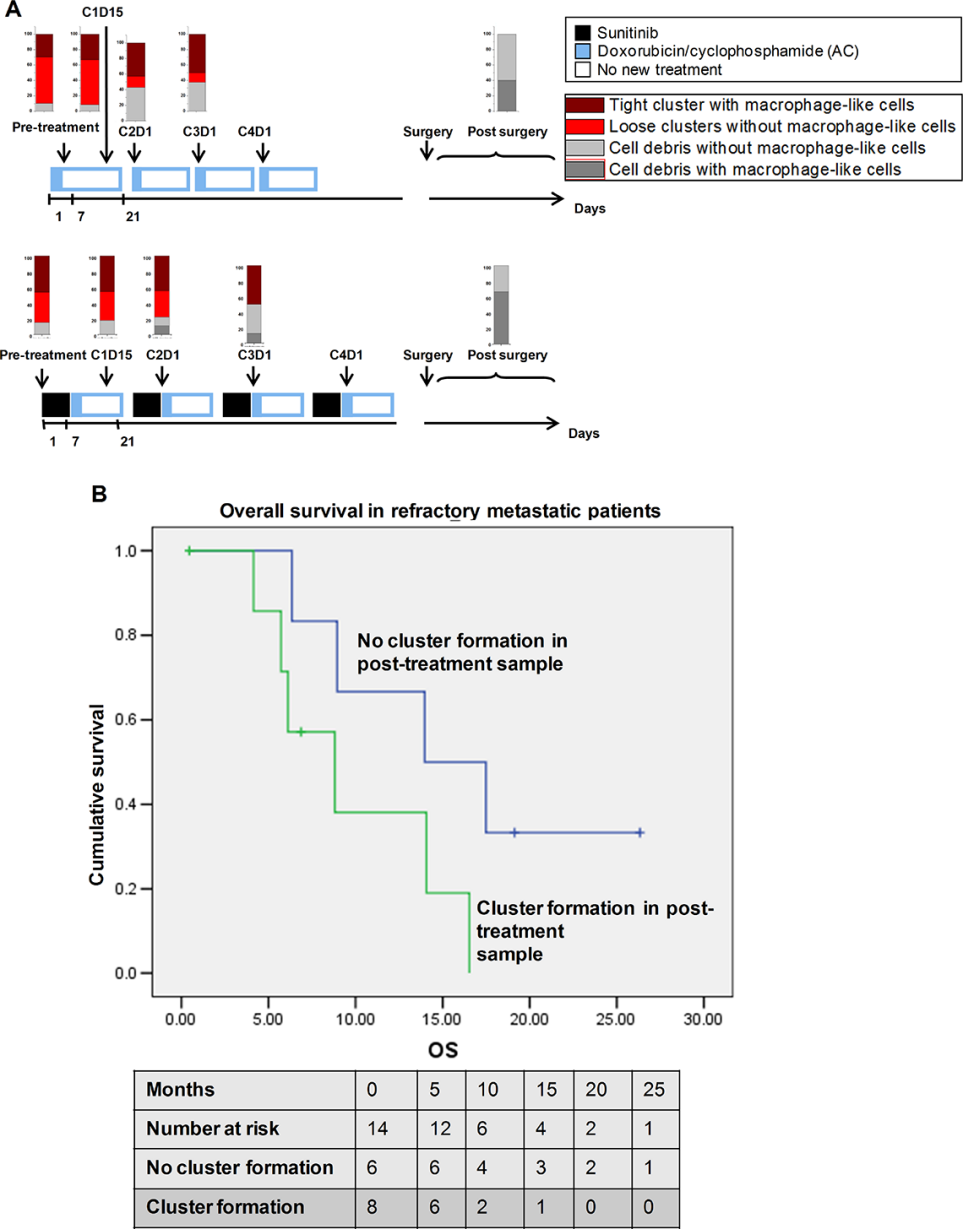
Clinical correlation of cluster formation with patient survival **A.** Treatment schedule for the patients (*n* = 31) receiving doxorubicin/cyclophosphamide (AC) with or without Sunitinib. Cluster formation is reduced during therapy cycles, reflecting response to chemotherapy for treatment efficacy. **B.** Comparison of overall survival in refractory metastatic patients who had or did not have cluster formation in the post-treatment sample (*n* = 14).

To further investigate this correlation between cluster formation and response, serial (4 or more) samples (a total of 90) were collected from 18 patients in Cohort P2A/B over a period of 12–16 weeks; this cohort comprised patients with early-stage breast cancer who had been treated with pre-operative doxorubicin/cyclophosphamide (AC) with or without Sunitinib as part of a clinical trial (Supplementary Table 1A, Figure 5A) and then undergone breast conserving surgery or mastectomy along with axillary lymph node clearance. We observed a progressive reduction in cluster formation in samples from patients who had undergone increasingly longer treatments. Clusters formed in 30/35 (85.7%) of pre-treatment and post-1-week Sunitinib pre-chemotherapy (post-sutent, pre AC) samples (Figure 5A). More of the post-chemotherapy samples (at least one AC cycle with or without Sunitinib) did not form clusters (38/80; 47.5%) as compared with pre-chemotherapy samples (5/35; 14.3%). All of the negative cultures from post-treatment samples generated cell debris either with or without residual blood cells (Figure 1, Supplementary Table 1A). Interestingly, none of 10 post-surgical samples after 12 weeks of neoadjuvant chemotherapy formed clusters.

To correlate these treatment responses with survival, samples were obtained from 22 patients with refractory metastatic disease (Cohort CTB, Supplementary Table 1B); these patients were enrolled when they presented with progressive disease since their last treatment regimen but before they commenced a new treatment regimen (chemotherapy, endocrine therapy or radiotherapy); blood was collected before and at 3–5 weeks after the new treatment regimen. Post-treatment samples were available from 14/22 patients, and we found an interesting correlation between cluster formation in these samples and clinical response and survival. Cluster formation from post-treatment samples was more common in patients with early radiological progressive disease (3/4, 75%) as compared with those with radiological responsive or stable disease (4/9, 44.4%; *p* = 0.308; Supplementary Table 6). One patient was unassessed at this point in time. Kaplan–Meier survival analysis showed cluster formation in the post-treatment sample correlated with shorter overall survival, with a mean overall survival of 9.8 months (95%CI, 5.9–13.7) for patients whose samples generated clusters as compared with a mean overall survival of 16.6 months (95%CI, 10.4–22.8; log rank *p*-value, 0.087) for patients whose samples did not yield clusters (Figure 5B). Because of the limited sample size, further Cox regression and adjusted analyses were not undertaken. However limited, these findings are intriguing in terms of showing a possible correlation between overall survival and cluster formation, and a larger set of clinical samples is currently being collated to validate these findings.

Finally, we attempted to evaluate the possibility of detecting cultured CTCs in early-stage cancer. The estimated relapse frequency for early-stage breast cancer in Singapore is 10%–20% for stage I, 30%–40% for stage II, and 50%–70% for stage III [43]. We analyzed 53 samples from 32 patients with early-stage breast cancer (stages IA–IIIC) who had undergone surgery, had no clinically measurable tumor, and were awaiting, undergoing or had just completed adjuvant chemotherapy (Supplementary Table 1D). Overall, 23/53 (43.4%) samples formed clusters. Clusters were more commonly observed in patients with pathological involvement of 4 or more lymph nodes (9/16 (56%)) as compared with those with 0–3 lymph nodes (14/37 (38%); *p* = 0.214). We also correlated cluster formation from cultured CTCs with time since surgery, and found that 10/20 (50%) samples taken shortly after surgery but before adjuvant chemotherapy formed clusters. For samples taken shortly after completing 3–6 months of adjuvant chemotherapy, cluster formation reduced to just 26% (6/23) but this rebounded to 70% (7/10) (*p* = 0.049) for samples obtained 1 year post-adjuvant chemotherapy. Among these ten samples cluster formation occurred in 2/4 (50%) patients with pT1N0M0 disease at later time points, as compared to 5/6 (83%) patients with a higher pathological stage of the disease (*p* = 0.260).

## DISCUSSION

Monitoring CTC levels may offer a vast improvement in the current diagnosis of cancer and may help determine the efficacy of selected therapeutic regimes. The copious technical approaches developed for the detection of CTCs [24–27, 37, 44] have thus far failed to retrieve an adequate number of viable cells for culture and characterization, and these methods also require a finicky, delicate or time-consuming procedure to do so. More recent approaches to culture CTCs suffer from low efficiencies (< 20%) [12, 13, 15] and the need for pre-enrichment steps that, paradoxically, result in the loss of CTC counts. Hence, there is still a need for a novel method with improved efficiency and without the need for prior enrichment for practical use in the clinic.

Here, we describe an effective method for the *in vitro* expansion of CTCs from patients with early stage, locally advanced, or metastatic breast cancers. We observed cell clusters comprising hundreds of cells by Day 14 of culture, comprising both ‘Small’ (≤ 25 μm) and ‘Large’ (> 25 μm) cell fractions (Supplementary Figure 2). Most of the Large cells were CD68+ with phagocytic activity and are likely to be macrophages (Supplementary Figure 4). Indeed, macrophage-like cells have been detected in blood samples from various cancer patients by others who have speculated a correlation between the presence of macrophages and the metastasis potential of CTCs [45]. We also detected other CD56+ blood cells that corresponded to NK cells, but could not detect megakaryocytes, monocytes, endothelial cells or MSCs or their lineages derivatives.

The Small cell fraction demonstrated a phenotype that was consistent with the standard definition of CTCs (pan-CK+/CD45-/Hoechst+ with a high N/C ratio [46]). Immunofluorescence labeling revealed that the majority of ‘Small’ cells were positive for Vimentin and Fascin, and had low levels of EpCAM and E-cadherin, further emphasizing their mesenchymal-like phenotypes (Figure 3). Results from RNA FISH, on the other hand, confirmed the presence of epithelial, epithelial–mesenchymal and fully mesenchymal phenotypes within these cultures, and the varied proportions of these three phenotypes did not appear to be related to the ER/PR/HER2 status of the tumor (Supplementary Figure 13). This heterogeneous expression of both epithelial and mesenchymal markers is consistent with previous reports by others on CTCs in breast [29], prostate [49, 50] and head and neck [51] cancers. We further used pooled samples to carry out DNA FISH for six genes previously reported to contribute to driver mutations in breast cancer: MYC, FGFR1, CCND1, HER2, TOP2A, and ZNF217 (Figure 4). Through this assay, we detected locus copy number increase or amplicons in 6/10 (40%) samples using single probes, and in another 21/27 (77.7%) samples using combined probes.

Although initial CTC counts may not reflect the potential of a sample to be cultured (due to cancer cell dormancy [47, 48]), it will be worth reviewing the relationship between cluster formation and initial CTC counts among a larger sample cohort. We showed that cultured cells could be transferred to Geltrex® or ultra-low adhesive dishes and grown as spheroids, withstanding multiple passages (Supplementary Figure 12). It is possible that these spheroids could then be maintained and used for *ex vivo* drug analysis, which has the potential to guide the selection of drugs for therapy. If an *ex vivo* drug analysis assay is validated and proven to be reliable, then the relatively short turnaround time (14 day culture) is feasible in clinical practice to guide drug selection in the advanced and neoadjuvant, and even in the adjuvant, settings. Future studies will be aimed at establishing correlations between spheroid formation, cancer stage and treatment time-points.

The maintenance of CTCs in culture after enrichment has been challenging [37]. Most CTC enrichment devices alter cell viability; possibly as a consequence of the lengthy procedures and the loss of potentially clonogenic CTCs. Few techniques have reported the enrichment of viable CTCs [44, 52]. Affinity-based devices further limit the detection of CTCs to various subpopulations due to the pre-requisite of enriching cells with certain epithelial markers (e.g., EpCAM) [27, 53] that may be down-regulated during EMT [53, 54]. Recent methods for the expansion of CTCs have been reported [12, 15], including the initial proof-of-concept study for CTC cultures by Zhang and colleagues [13]. However, these methods are currently still unable to generate cultures efficiently. Emerging inertial microfluidic devices may help to solve this issue [28, 55, 56].

Our culture system enabled us to perform various characterizations with primary cells after a short-term culture of 14 days. This is more advantageous than using cell lines, since prolonged culture and multiple passages often lead to phenotypes that are no longer representative of the original tumor in terms of the cell's epigenetics and gene expression [57, 58]. Our method also requires as little as 2.5 ml of blood per 60-mm dish to yield tens of thousands of cells within two weeks, and provides a risk-free approach to CTC harvesting and expansion, with no adverse effects to cell viability, concomitant with the disappearance of WBCs and other non-malignant circulating cells. Our microwell geometry favors the expansion of CTCs by reducing cell–substrate contact through the use of a non-adhesive plastic substrate. We believe that the curvature of the tapered microwells presents a topography that encourages cell clustering, generating a suitable microenvironmental niche that resembles the tumor microenvironment; this enables CTCs and blood cells to cluster under hypoxic conditions with limited nutrients, as *in vivo*. Cultures are sheltered within microwells and experience minimal disturbance during media changes, reducing shear stress and cell loss to allow for subsequent expansion. These culture conditions and minimal starting material provide an opportunity for the deep characterization of CTCs, specific to each patient, and a technique with these benefits will be of paramount importance for optimal applications in the clinic, which often require repeated sampling.

The proliferative potential of these cultured cells varies between samples and critically depends on the type and duration of therapy administered to the patients. The absence of cell cluster formation in the microwells from post-treatment samples may reflect treatment efficacy [31], and may possibly reflect a lack of cancer-initiating cells following one or several cycles of treatment. The clusters described here may arise from either single CTCs or microemboli [28, 59]. Interestingly, we also observed that some of the blood samples that did not initially contain detectable CK+ CTCs were later positive at Day 14 in culture (Supplementary Table 2). This may be a result of the proliferation of very few CTCs with heightened survival characteristics. A previous attempt by Pizon et al. to expand breast epithelial CTCs into spheroids also observed a similar increase in spheroid formation from patients with more aggressive tumors [60], despite variations in the enrichment technique (which selects for epithelial cells under normoxia). Hence, such studies strengthen the hypothesis that CTC cultures may correlate with disease severity.

Our results lend to the hypothesis that systemic therapy affects cluster formation and that a persistence of the ability to cluster may reflect therapy resistance. Cluster formation was seen progressively less frequently in samples with a longer duration since systemic therapy. Strikingly, none of 10 post-surgical samples (after completing neoadjuvant chemotherapy) in the P2A/B cohort demonstrated cluster formation (Supplementary Table 1A). An early loss of cluster formation in this cohort occurred in 1 of 2 patients; this patient achieved pathological complete response after 12 weeks of neoadjuvant chemotherapy. Also, samples from patients with ILC had lower tendencies to form clusters, which could be due to the E-cadherin status of the tumors. However, our sample size of ILC is too small in this study for us to draw firm conclusions about the relationship between ILC, chemotherapy response and CTC cluster formation. Persistent cluster formation in post-treatment samples taken from refractory metastatic disease patients at a time-point 3–5 weeks after treatment (CTB cohort; Supplementary Table 1B)) correlated with early disease progression (within 12 weeks of treatment) and shorter overall survival. We also assessed cluster formation in a small cohort of patients with early-stage breast cancer (CES cohort; Supplementary Table 1D); these patients had undergone surgery and were at sufficient risk clinically to warrant subsequent adjuvant chemotherapy. We were intrigued to find cluster formation in about half of the patients who had no clinically measurable disease, and noted that cluster formation was more frequent in patients with higher lymph node involvement (≥ 4). The positivity rate of 39% (7/18) for CTC cluster formation in post-surgical specimens from early stage, pathologically node-negative breast cancer patients reported in this study is much higher than the EpCAM-independent CK-19 mRNA positivity rate reported in a study involving node-negative breast cancer (21.6%) [61]. It will be relevant to investigate more closely the implications of cluster formation in samples from node-negative patients. Interestingly, cluster frequency was reduced from 50% in immediate post-surgical samples to 26% in samples taken after 3–6 months of adjuvant chemotherapy. Since we already see a tendency for clusters to form or re-appear in samples from node-positive patients at 1 year after adjuvant chemotherapy, it will be important to ascertain if there is any correlation between adjuvant chemotherapy and the long-term outcomes for patients in a larger cohort (using serial samples).

The current methods to evaluate response to chemotherapy in the neoadjuvant setting suffer from poor correlation between clinical response and the more relevant endpoint of pathological complete response. A reliable early test to predict pathological complete response is highly desirable and may permit adaptive clinical trials to be conducted in the neoadjuvant setting. The patients recruited in this study are heterogeneous in terms of treatment choice, metastatic sites, and other demographic characteristics. Future studies involving a more homogeneous study population may lead to better understanding of the effects of treatment on cluster formation and the correlation between presence and persistence of clusters with patient survival.

Overall, we established a method for the *in vitro* expansion of CTCs using a significant number of blood samples (*n* = 226) from patients with early stage, locally advanced or metastatic breast cancers. Blood samples from healthy subjects (*n* = 16) were also cultured in the assay, which consisted of laser-ablated microwells. We investigated the clinical impact of *in vitro* CTC-clustering as a prognostic and predictive tool for therapy response and explored the phenotypic and genotypic characteristics of the heterogeneous CTCs after 2 weeks in culture. Although we cannot yet confirm the clinical utility (due to small sample cohort and lack of long-term outcomes), this method detects more patients with CTCs than any other method described to date, and can even induce the expansion of CTCs in apparently initially negative samples. We believe that the method is exploitable for the study of drug responses *in vitro* for locally advanced or metastatic cancer treatment.

## MATERIALS AND METHODS

### Preparation of culture assay

The culture assays were fabricated using uncoated 60-mm petri dishes (Becton Dickinson, Franklin Lakes, NJ) and microwells were patterned using a commercial air-cooled 10.6-μm CO_2_ laser engraving/cutting system at 10% speed and 50% power (VLS-2.30, Universal Laser System Inc., Scottsdale, AZ). Typically, each 60-mm patterned dish contains 1350 ellipsoid-shaped tapered wells (Figure 1A). The dimensions of the microwell entrance (225 μm × 145 μm) are outlined in Figure 1B and 1D, and the overall structure of the microwell can be demonstrated with a PDMS replica (Figure 1C) (Polydimethylsiloxane) (1:10 ratio (Sylgard 184, Dow Corning, USA)) prepared and demolded, as previously described [27]. Wells were rinsed and incubated with 70% ethanol for at least 15 min for sterilization.

### Sample preparation

Blood samples were obtained from 92 breast cancer patients (Supplementary Tables 1A–1D). This study was approved by our institutional review board and local ethics committee (DSRB Reference 2012/00105, 2012/00979, 2010/00270, 2010/00691). Blood samples were obtained from patients enrolled into four different studies, including two neoadjuvant studies (doxorubicin/cyclophosphamide with or without Sunitinib [P2A/2B cohort]; paclitaxel/carboplatin/lapatinib [PCL cohort]), one study of refractory patients treated with various treatment regimens (CTB cohort), and one early-stage breast cancer study after definitive breast cancer surgery (CES cohort). Sixteen healthy volunteers (DSRB-2013/00542) were also recruited to provide control blood samples for validation of culture assay specificity. All patients gave their informed consent for inclusion in this study. Clinicopathological information was recorded for each patient. Samples were collected from each patient once in a single draw, either before or after treatment, or at several time-points over their treatment period. All blood specimens were collected in sterile EDTA-coated vacutainer tubes (*Becton Dickinson*) and kept on ice (Figure 1E). The demographic and clinical treatment characteristics for the three cohorts of 60 patients with clinically measurable tumors (i.e., excluding the CES cohort) are summarized in Table 1.

**Table 1:** Patients with clinically measurable tumors

Demographic or clinical characteristics	No. of patients involved (*n* = 60)	
		%
**Age (years)**		
Median	47.5	
Range	33–78	
**Race**		
Chinese	38	63.3
Indian	5	8.3
Malay	11	18.3
Others	6	10
**Histology**		
IDC	49	81.7
ILC or IDC with lobular features	5	8.3
Others	6	10
**Tumor grade**		
1	3	5
2	18	30
3	35	58.3
Not specified	4	6.7
**Metastatic disease**		
Yes	25	41.7
No	35	58.3
**AJCC stage**		
I	0	0
II	16	26.7
III	19	31.7
IV	25	41.7
**ER status**		
Negative	21	35
Positive	39	65
**PR status**		
Negative	18	30
Positive	42	70
**HER2 status**		
Negative	47	78.3
Positive	13	21.7
**Treatment regimen**		
AC	15	25
AC+Sunitinib	16	26.7
Paclitaxel/carboplatin/lapatinib	7	11.7
Others	22	36.7

IDC = invasive ductal carcinoma; ILC = invasive lobular carcinoma; AJCC, American Joint Committee on Cancer; ER = estrogen; PR = progesterone; HER2 = human epidermal growth factor receptor 2; AC = doxorubicin/cyclophosphamide.

Samples were processed within 10 h after withdrawal to reduce blood clotting and maintain cell viability. Whole blood was lysed with RBC lysis buffer (Life Technologies, Carlsbad, CA) for 3–5 min with gentle mixing, centrifuged to remove plasma and lysed RBC fragments, and then washed once with sterile phosphate-buffered saline (PBS). Remnants of the buffer were removed quickly to reduce cell damage upon prolonged exposure. Nucleated cells were re-suspended in fresh, high-glucose Dulbecco's modified Eagle's medium (DMEM) supplemented with 10% fetal bovine serum (FBS) and 1% penicillin-streptomycin (all from Invitrogen, Carlsbad, CA). Each processed sample (10 ml of whole blood) was split into four and each 2.5 ml sample seeded into separate 60-mm patterned dishes.

### Characterization of clusters in blood cultures

Samples were kept at 37°C in 5% (v/v) CO_2_ and 1% O_2_ under humidified conditions. The culture medium was replaced every 48–72 h with minimal disturbance to the microwell clusters to avoid cell loss. Clusters were dissociated with pipetting following incubation for a maximum of 3 min at 37°C with 0.01% trypsin and 5.3 mM EDTA (Lonza, Basel, Switzerland) solution in PBS.

Cultures were maintained for 2–8 weeks, imaged on Days 0, 8, 14 and 21 with phase contrast microscopy and analyzed with ImageJ (NIH, Bethesda, MD). The mean diameters of the structures are the average maximum and minimum dimensions along the same 2D plane of each aggregate.

### Cell sorting with spiral inertia microfluidic biochip

Cultured cells used for histopathological characterization by PAP staining (see below) were sorted for better contrast and comparison of morphological differences between populations of different cell sizes. Cells were trypsinized, re-suspended in 1 ml of media within a 10-ml syringe and pumped at 100 μl/min through a PDMS spiral inertia microfluidic biochip [27]. Cells were administered together with the sheath fluid containing PBS at 800 μl/min to obtain size-sorted cell populations for further analysis. Sorted cells were concentrated via centrifugation.

### Immunophenotyping of cells

Cells were fixed with 4% paraformaldehyde (PFA) (Sigma-Aldrich, St Louis, MO) and permeabilized with 0.1% Triton X-100 (Thermo Fisher Scientific, San José, CA). Fluorescence microscopy was performed using primary antibodies (Supplementary Table 4) and Dylight 488 or 594 secondary antibodies (Abcam, Cambridge, United Kingdom) and counterstained with Hoechst dye (Invitrogen) (See Supplementary Methods).

### Immunophenotyping of cells via cytospots

CTC cultures or control cell lines (ESCs, MSCs, macrophages, endothelial cells, MCF-7 and MDA-MB-231) were trypsinized, concentrated in PBS, and prepared as cytospots, as described in the Supplementary Methods using a Cytospin 4 cytocentrifuge (Thermo Fisher Scientific). Slides were fixed and permeabilized as above and incubated with a range of primary antibodies (Supplementary Table 4), followed by appropriate Dylight 488 or 594 secondary antibodies (Abcam) and counterstained with Hoechst dye (Invitrogen). Antibody specificity was validated with negative controls using either MCF7, MDA-MB-231 or lysed blood samples at Day 0 before culture. Putative CTCs were identified as CK+/CD45-/Hoechst+ cells.

### Enumeration of CTCs

Ten samples were analyzed to quantify CK+ sub-populations (Figure 2B, Supplementary Table 2). For Day 0 samples, 100 μl of freshly lysed samples were fixed onto coated slides using cytospinning (as described above). For Day 8, 14 and 21 cultures, cells were harvested and fixed onto slides (as described above). Slides were then stained with pan-cytokeratin-FITC, CD45-APC and Hoechst. The entire cytospot was imaged to detect positive cells, and the CK+/CD45-/Hoechst+ cell counts/ml were estimated (Supplementary Table 2).

### Histological staining and imaging

Cytospots were viewed after PAP staining at the Advanced Molecular Pathology Laboratory at IMCB, Singapore. Diff-QUIK Romanowski staining was performed at the Pathology Department of the National University Hospital, Singapore. Imaging was performed with confocal (Olympus Fluoview FV1000, USA) or epifluorescence (Nikon, Japan) microscopy.

### DNA and RNA Fluorescence *in situ* hybridization (FISH)

Cytospots were fixed with acetic acid/methanol (Sigma-Aldrich) in a 1:3 ratio, added drop-wise to the cell spot at room temperature, and dehydrated through a graded ethanol series (80%, 90%, and 100%). DNA FISH and RNA FISH were performed as outlined in the Supplementary Methods.

### Spheroidogenic assays

Cultures at Day 14 were harvested, separated into single cells and mixed with Geltrex® (Invitrogen, cat. no. 12760–013) at recommended concentrations in wells of 16-well glass chamber slides (Lab-Tek Products, Miles Laboratories, Naperville, IL). Cultures were maintained under optimal conditions for 1 week, fixed and stained with TRITC-phalloidin (1:1000, Sigma-Aldrich) and Hoechst for 1 h. Chambers were washed and imaged with a confocal microscope to obtain z-stacks of 1 μm. Alternatively, spheroids were obtained by transferring Day 14 cultured cells to ultra-low adhesive dishes (Cat No; 3473 or 3473; Corning Inc., Corning, NY) and maintained for 10 days in advanced DMEM/F12, reduced-serum medium (1:1) (Gibco, Life Technologies, Carlsbad, CA) under normoxia prior to subsequent transfer to amplify the spheroids.

### Statistical analysis

The *χ*^2^ test (when the sample size was small) was used to evaluate associations between categorical variables and spheroid formation. A two-way analysis of variance (ANOVA) was employed using Microsoft® Excel® (Redmond, WA) to analyze the flow cytometry data.

## SUPPLEMENTARY METHODS FIGURES AND TABLES











## Abbreviations

CTC: circulating tumor cell
EMT: epithelial-mesenchymal transition
N/C: nuclear/cytoplasmic ratio
FISH: fluorescence *in situ* hybridization
CSC: cancer stem cell
ES: embryonic stem
EGFR: epidermal growth factor receptor
CK: cytokeratin
EM: Epithelial-Mesenchymal
E: Epithelial
M: Mesenchymal
NK: natural killer cells
MSC: mesenchymal stem cells
ER: estrogen receptor
PR: progesterone receptor
HER2: human epidermal growth factor receptor 2
PAP: Papanicolaou
AC: doxorubicin/cyclophosphamide
DMEM: Dulbecco's modified Eagle's medium
FBS: fetal bovine serum
BSA: bovine serum albumin
PBS: phosphate-buffered saline
N: Negative
Y: Positive
C: Chinese
M: Malay
I: Indian
O: Others
IDC: invasive ductal carcinoma
ILC: invasive lobular carcinoma
FAC: 5-fluouracil/doxorubicin/cyclophosphamide
