# Supplementary material for: Short-term expansion of breast circulating cancer cells predicts response to anti-cancer therapy
Source: Oncotarget. 2015 May 6;6(17):15578–93. doi: 10.18632/oncotarget.3903 (PMC4558172; doi:10.18632/oncotarget.3903)
Supplement: Supplementary file 1 [file oncotarget-06-15578-s001.pdf]

SUPPLEMENTARY METHODS

Immunophenotyping of cells in culture

Media was removed without disturbing the clusters, and cells were fixed with 4% paraformaldehyde (PFA) (Sigma-Aldrich, St Louis, MO) at room temperature for 10 min. Fixed cells were then permeabilized with 0.1% Triton X-100 (Thermo Fisher Scientific, San José, CA) in PBS on ice for 1 min. Primary antibodies (Supplementary Table 4) were diluted in 200 µl of PBS supplemented with 2% bovine serum albumin (BSA) and added to the patterned dishes. Dishes were incubated for at least 1 h on ice. For secondary labeling, dishes were washed gently and incubated for ~1 h on ice with respective Dylight 488 or 594 secondary antibodies (Abcam, Cambridge, United Kingdom) and counterstained with Hoechst dye (Invitrogen).

Immunophenotyping of cells via cytospots

CTC cultures or control cell lines (embryonic stem cells (ESCs), mesenchymal stem cells (MSCs), macrophages, endothelial cells, MCF-7 and MDA-MB-231) were trypsinized and concentrated in PBS. Cytospots were generated with 100 µl of cell suspension at 600 rpm for 5 min on frosted slides (Thermo Fisher Scientific) using a Cytospin 4 cytocentrifuge (Thermo Fisher Scientific). Slides were fixed and permeabilized as previously described for cells and incubated with a range of primary antibodies (Supplementary Table 4) for 30 min at 4°C. These antibodies were validated for their specificity with negative controls using either MCF7, MDA-MB-231 or lysed blood samples at Day 0 before culture. Slides were washed and incubated for another 30 min on ice with respective Dylight 488 or 594 secondary antibodies (Abcam, Cambridge, United Kingdom) and counterstained with Hoechst dye (Invitrogen). Putative CTCs were identified as CK+/CD45-/Hoechst+ cells. Antibody staining was conducted independently of pan-CK antibody staining to prevent cross-reactivity. Beta-galactosidase staining was performed at pH 6 to identify senescent cells.

DNA FISH

Dehydrated slides were treated with 4 mg/ml RNase (Sigma-Aldrich) in PBS for 45 min at 37°C, washed in 1× PBS/0.2% Tween-20 thrice and denatured with a 70% formamide/2×saline sodium citrate (SCC) (Sigma-Aldrich) solution for 10 min at 80°C. The slides were then dehydrated via another round of ice-cold graded ethanol series. Slides maintained at 42°C were then hybridized with probes (Supplementary Table 5), and denatured by incubation at 74°C for 5 min. Slides were sealed with rubber cement and kept at 42°C in dark and humid conditions for 16 h. Hybridized slides were washed with 50% formamide/2×SSC, followed by 2×SSC, both at 42°C with shaking, and then counterstained with Vectashield® mounting medium containing 4,'6'-diaminido-2-phenylindole (DAPI) (Vector Laboratories, Burlingame, CA) prior to mounting with a glass coverslip (Thermo Fisher Scientific). Slides were imaged with an epifluorescence microscope (Nikon, Japan) and z-stacks for each channel were obtained at 63 ×. ImageJ (NIH, Bethesda, MD) was used to achieve the projected image (maximum intensity) and the fluorescence signal dots were enumerated accordingly.

RNA FISH

Cytospots were prepared as described above. Cells were then permeabilized and incubated with customized probes obtained from iDNA (Affymetrix, Santa Clara, CA) (Supplementary Table 3) using the Quantigene kit (Affymetrix) according to recommended protocols. Slides were sealed with rubber cement and kept at 42°C in dark and humid conditions for 16 h. Hybridized slides were treated with components of the kit as instructed and counterstained with Vectashield® mounting medium containing DAPI (Vector Laboratories) prior to mounting with a glass coverslip (Thermo Fisher Scientific). Probes were selected using breast cancer expression profiling databases to identify the most differentially expressed genes based on their highest and lowest EMT scores [1–2]. All probes were added to the same treated slide, incubated overnight, washed and amplified according to the recommended protocol. Slides were then counter-stained with DAPI before mounting. Slides were imaged as described for DNA FISH.

Phagocytosis assay for macrophages

Fluorescein-labelled polystyrene microbeads (1 µm) were added at high densities to a culture dish at Day 14 of culture. Beads were incubated with the CTC cultures under optimal conditions as described for the 24-h timepoint. Dishes were then washed with PBS, fixed with 4% PFA as described and imaged with confocal microscope at 20 × magnification.

REFERENCES

1. Akalay I, Janji B, Hasmim M, Noman MZ, Andre F, De Cremoux P, Bertheau P, Badoual C, Vielh P, Larsen AK, Sabbah M, Tan TZ, Keira JH, Hung NT, Thiery JP, Mami-Chouaib F, et al. Epithelial-to-mesenchymal transition and autophagy induction in breast carcinoma promote escape from T-cell-mediated lysis. Cancer Res. 2013; 73:2418–2427.
2. Tan TZ, Miow QH, Miki Y, Noda T, Mori S, Huang RY, Thiery JP. Epithelial-mesenchymal transition spectrum quantification and its efficacy in deciphering survival and drug responses of cancer patients. EMBO molecular medicine. 2014; 6:1279–1293.

SUPPLEMENTARY FIGURES AND TABLES

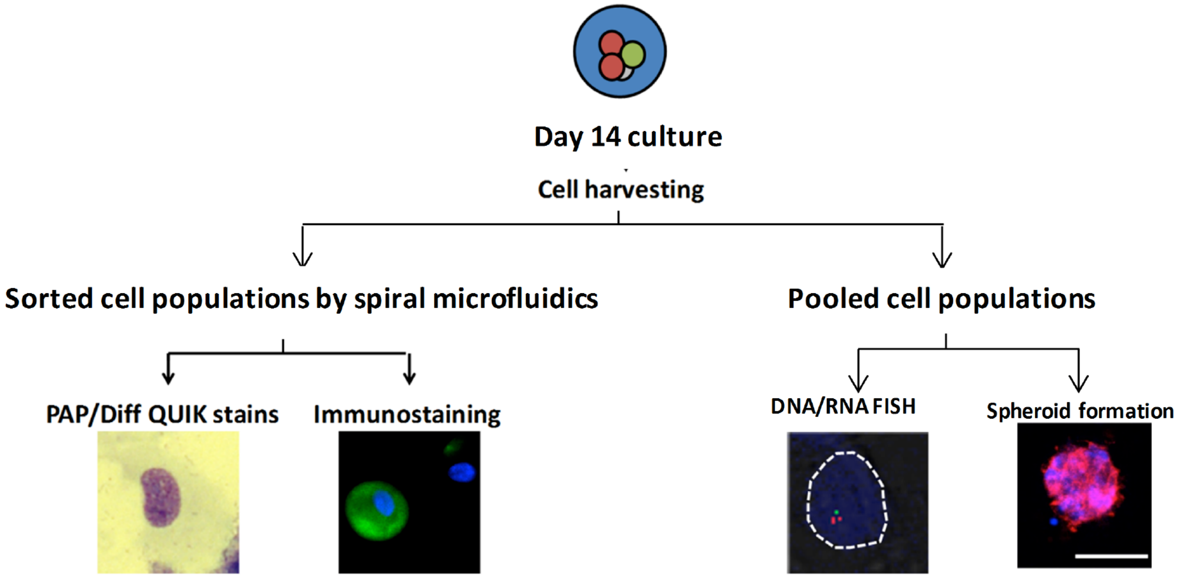

Supplementary Figure 1: Flow chart illustrating the experiments involved in this study.

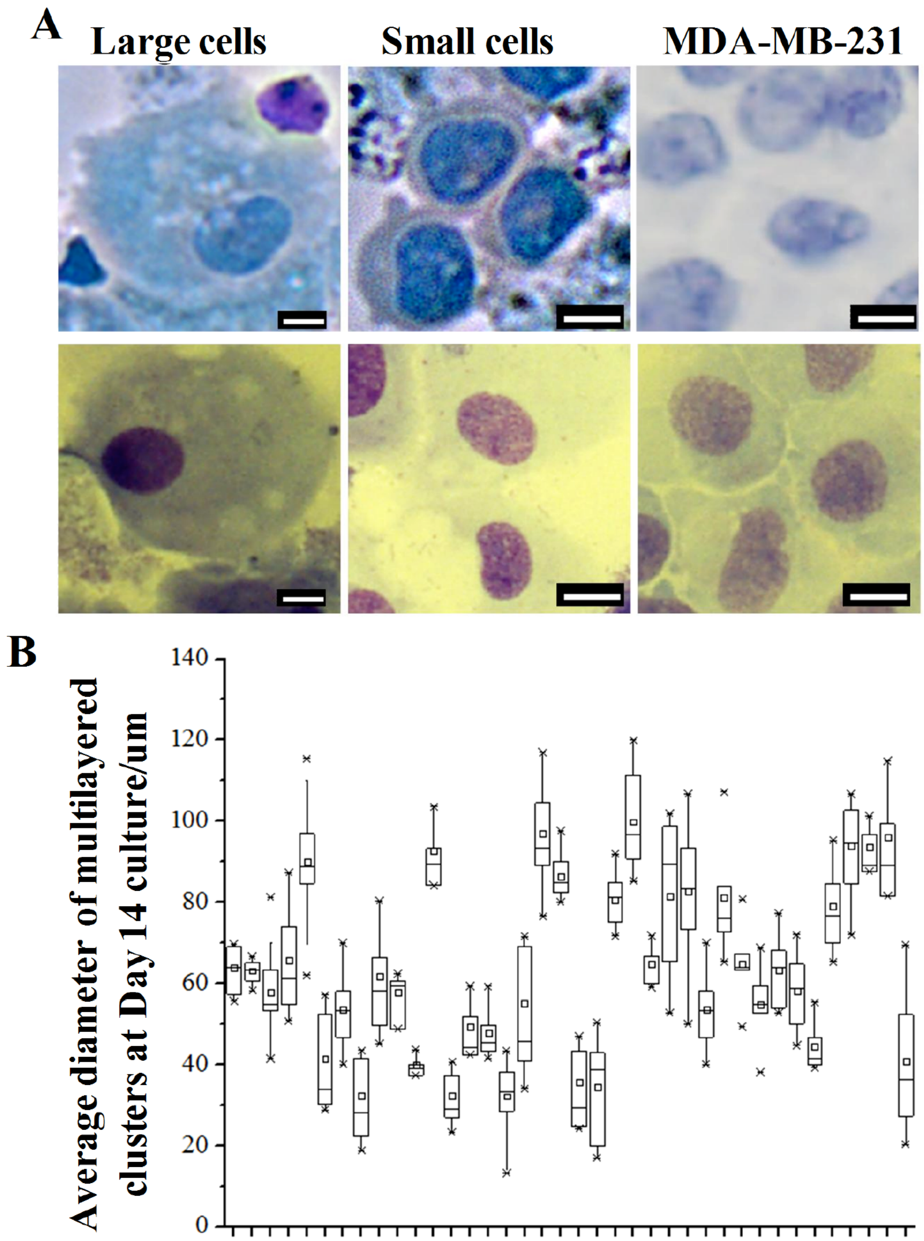

Supplementary Figure 2: Heterogeneity of cultured CTCs. A. Histopathology (*Papanicolaou* (PAP) and DIFF QUIK staining) of sorted cultured cells. Reddish-purple cells are erythrocyte 'ghosts'. Scale bar, 10 µm. **B.** Box plot demonstrating range of diameters of clusters at Day 14.

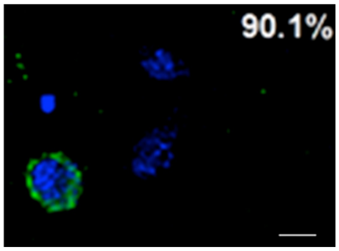

Supplementary Figure 3: Senescence assay. Beta-galactosidase staining (green) at pH 6 to determine the proportion of cells undergoing senescence (~9.9%). Scale bar, 20 µm.

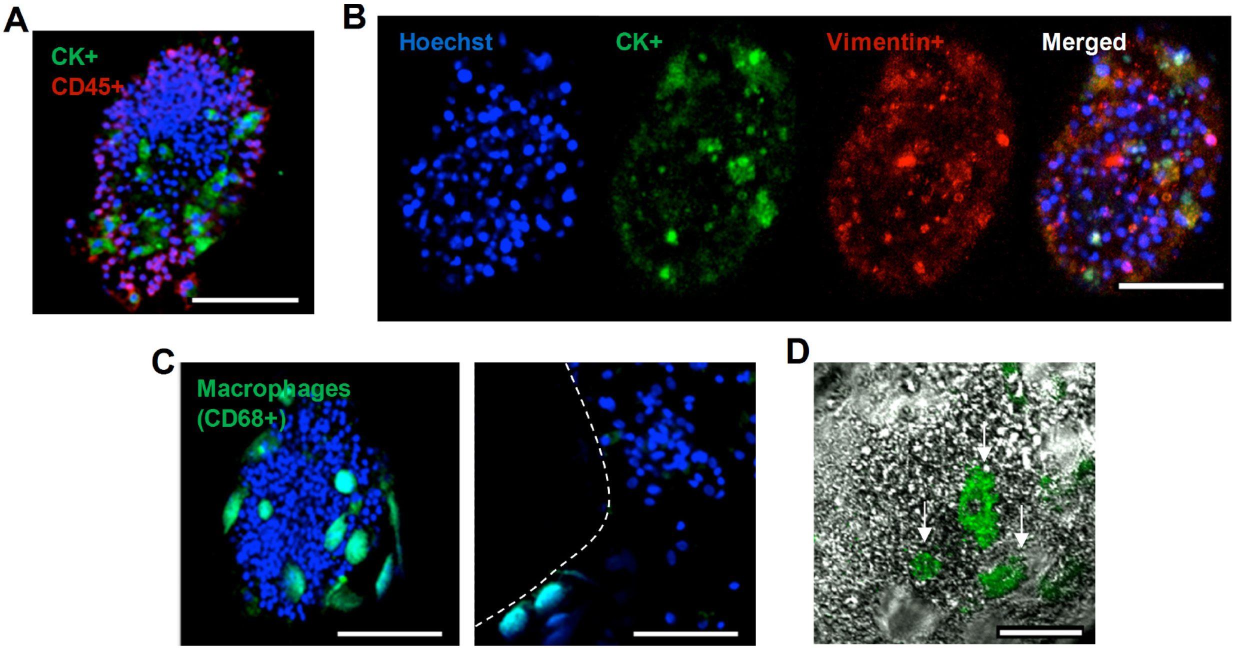

Supplementary Figure 4: Protein and gene expression of Day 14 cultures. A. *In situ* staining of Hoechst (blue), pan-CK-FITC and CD45-APC. CD45+ cells (< 15 µm) were generally localized at the periphery whereas CK+ cells (> 15 µm) were scattered throughout the cluster. Scale bar, 100 µm. **B.** *In situ* staining of Hoechst (blue), pan-CK (green) and Vimentin (red). High Vimentin expression was observed in most cells. Scale bar, 100 µm. **C.** *In situ* staining of Hoechst (blue) and CD68 (green) demonstrated the presence of macrophages within the cluster (left) and outside of the microwells (right). White dotted lines mark the boundary of the microwell. CD68 + cells seemed to correspond with the 'Large' cell fraction (> 25 µm). Scale bar, 100 µm. **D.** Differential interference contrast (DIC) imaging of Day 14 cultures after incubation with 1 µm fluorescein polystyrene microbeads for 24 h. Only 'Large' cells (white arrows) demonstrated uptake of fluorescence microbeads. Scale bar, 20 µm.

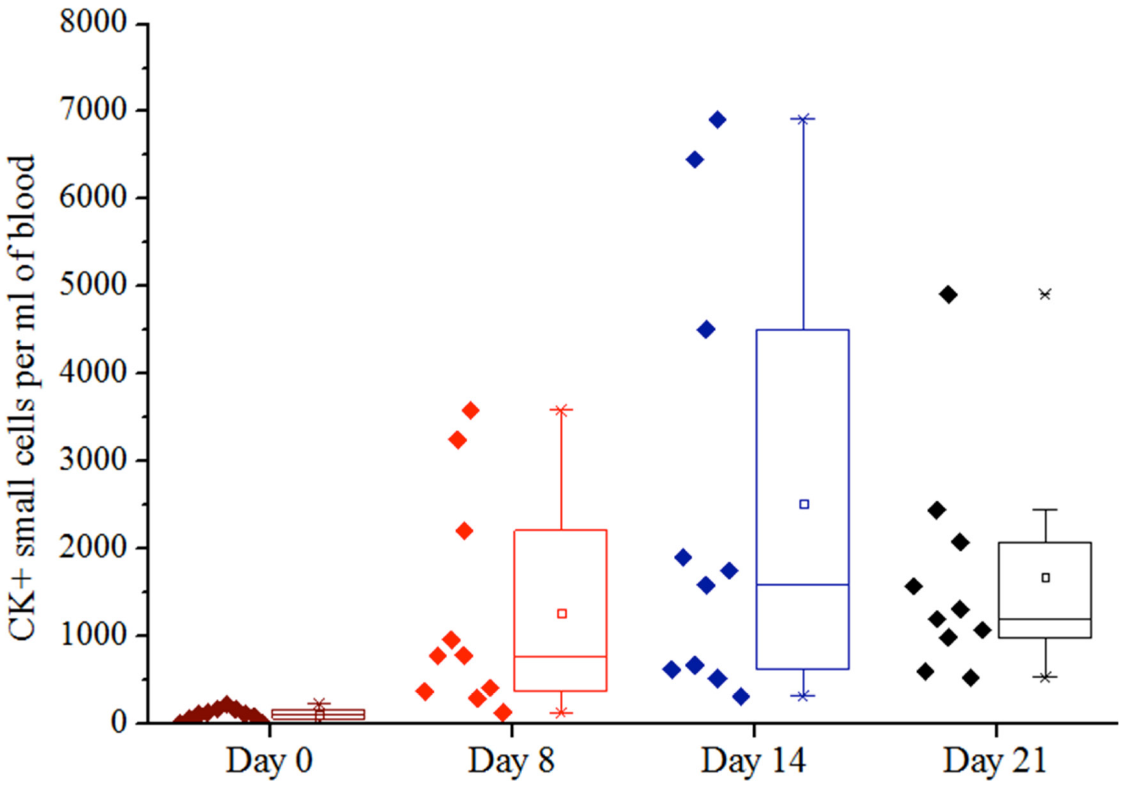

Supplementary Figure 5: Statistical box plot illustrating the CK+ small cell counts per ml of blood cultured at different time points (Days 0, 8, 14 and 21).

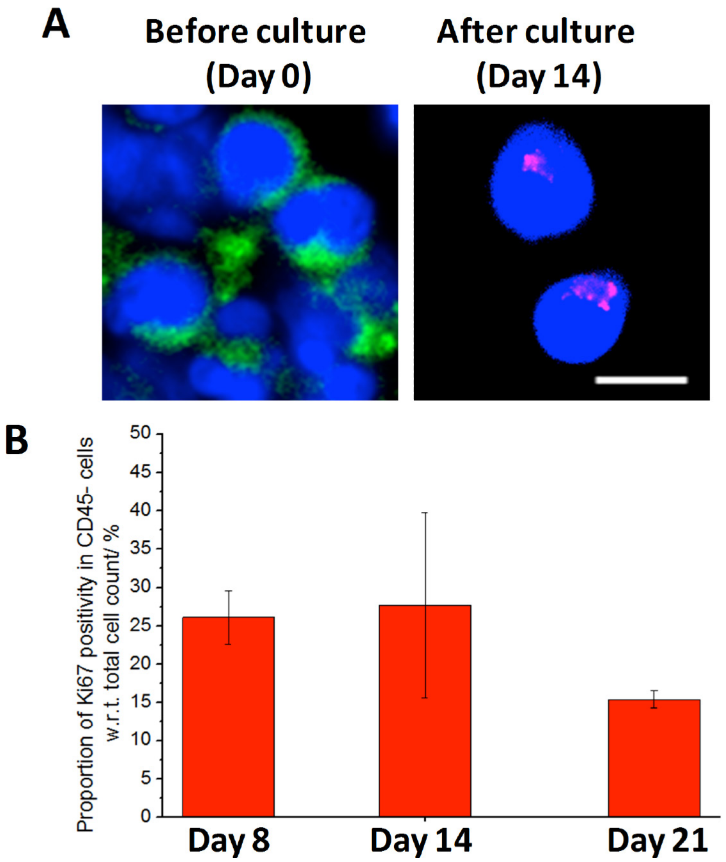

Supplementary Figure 6: Ki67 staining of cultures at different time-points. A. (Left) Immunostaining of samples at Day 0 before culture, using Hoechst, Ki67 primary antibodies (followed by 594 Dylight secondary antibodies) and CD45-FITC conjugated antibodies. (Right) Immunostaining of samples at Day 14 after culture. Scale bar is 10 µm. **B.** Graph representing the proportion of Ki67-positive/CD45-negative cells in culture at various time-points (Day 8, 14 and 21). The highest proportion of Ki67 cells can be usually detected in cultures at Day 14. w.r.t, with respect to.

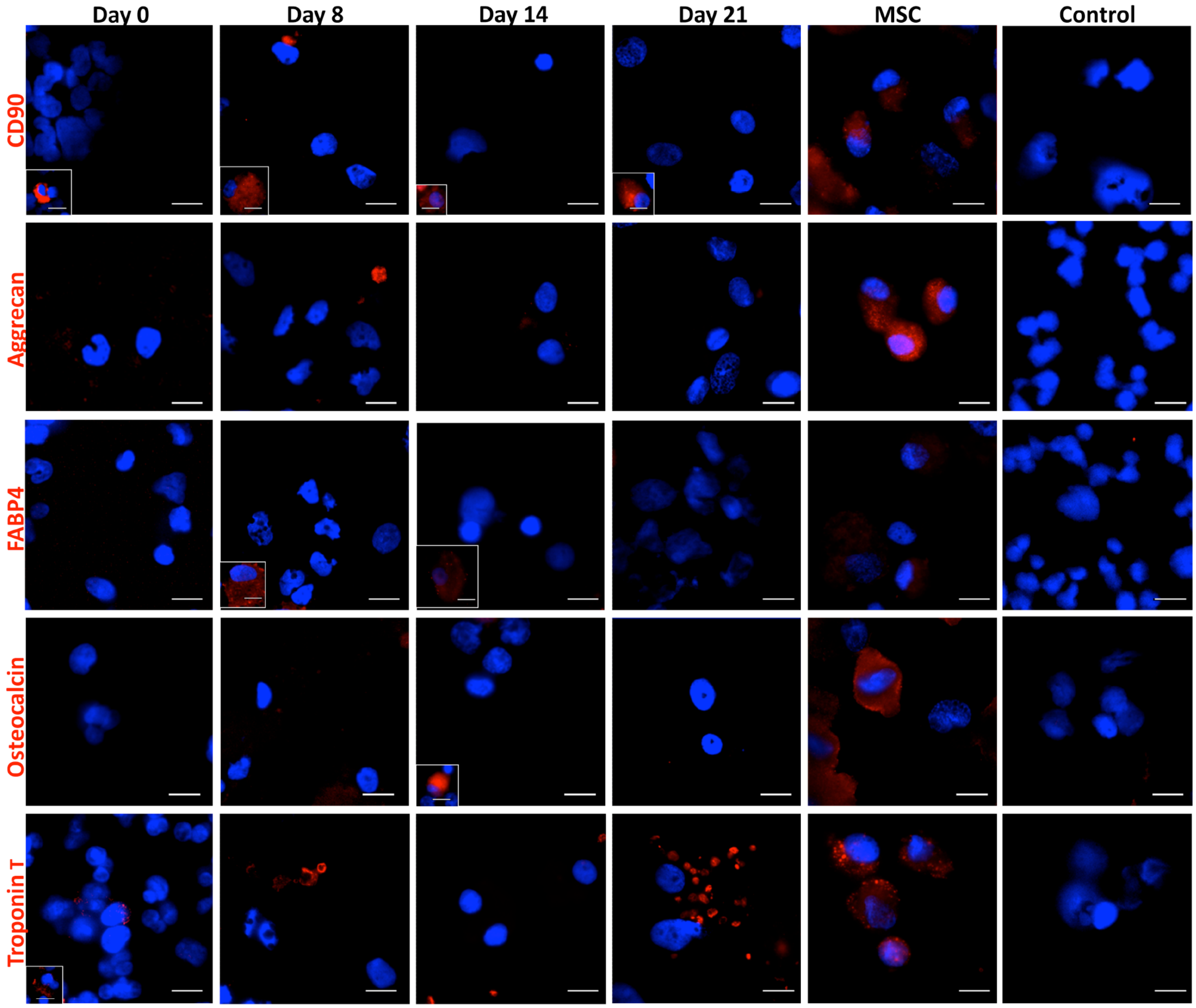

Supplementary Figure 7: Determination of MSC-associated sub-populations in culture. Immunostaining of cultured cells at different time points (Days 0, 8, 14 and 21) for MSC (CD90, Aggrecan, FABP4, Osteocalcin and Troponin T)-associated biomarkers. Cultures are mostly negative for MSC-associated biomarkers. Negative controls for the antibodies are provided in the last column using blood cells from Day 0 lysed blood. Boxed images (marked in white) provide examples of a distinct minority phenotype from the majority of cells. Scale bar, 20 µm.

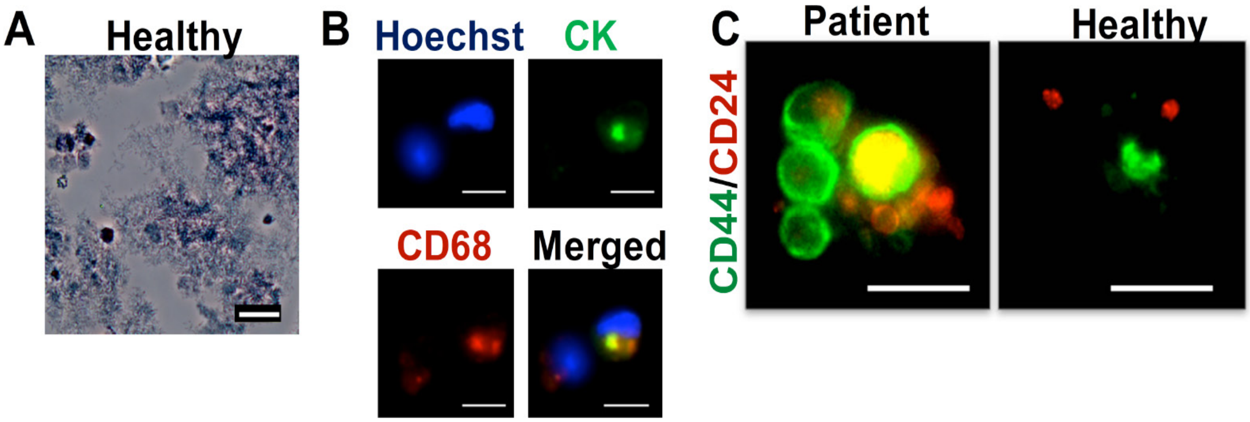

Supplementary Figure 8: Culture of healthy blood samples as a negative control. A. Non-proliferative cultures of healthy blood samples contain only cell debris (PAP staining). Scale bar is 10 μm. **B.** Immunostaining of Hoechst (blue), pan-cytokeratin (FITC) and CD68 (594) antibodies with cells obtained from healthy cultures. Most CK+/Hoechst+ cells are also CD68+, confirming the presence of blood cells. Scale bar is 10 μm. **C.** Immunostaining of CD44-FITC/CD24-APC for cultured cells from blood samples of patient and healthy donors. Cultures from healthy samples yielded only cell debris, with the marked absence of CD44+ cells. Scale bar is 100 μm.

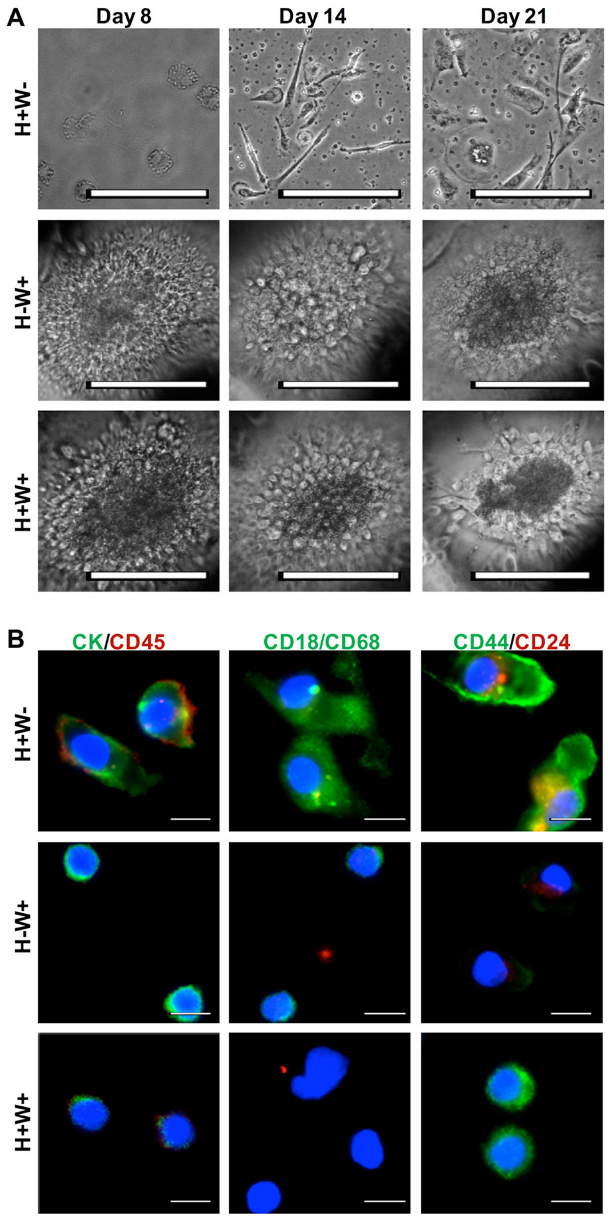

Supplementary Figure 9: Effects of oxygen level (Hypoxia = H+; Normoxia = H–) and topography (Microwell: W+; Flat substrate: W–) on resultant cell population distribution in culture. A. Time-lapse phase contrast images of cultures within microwells. 'Large' macrophage-like cells were present under all conditions. Scale bar, 100 µm. **B.** Immunostaining (CK-FITC/CD45-APC, CD68/CD18-488, and CD44-FITC /CD24-APC) of cells derived from cultures maintained under different conditions. CK/CD45 double-positive cells were most frequently observed in H+W- cultures. Scale bar, 20 µm.

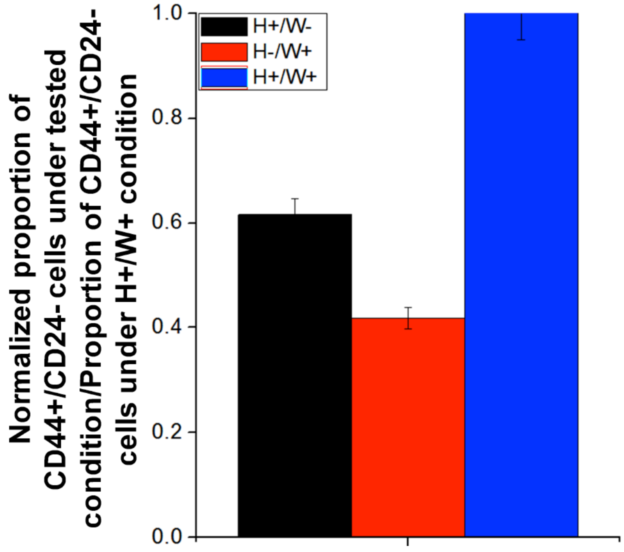

Supplementary Figure 10: Normalized proportion of CD44+/CD24- cells at Day 14 under different culture conditions. Percentage values were normalized to the percentage of CD44+/CD24- population detected with culture under H+/W+ condition. (Hypoxia = H+; Normoxia = H−) (Microwell: W+; Flat substrate: W−).

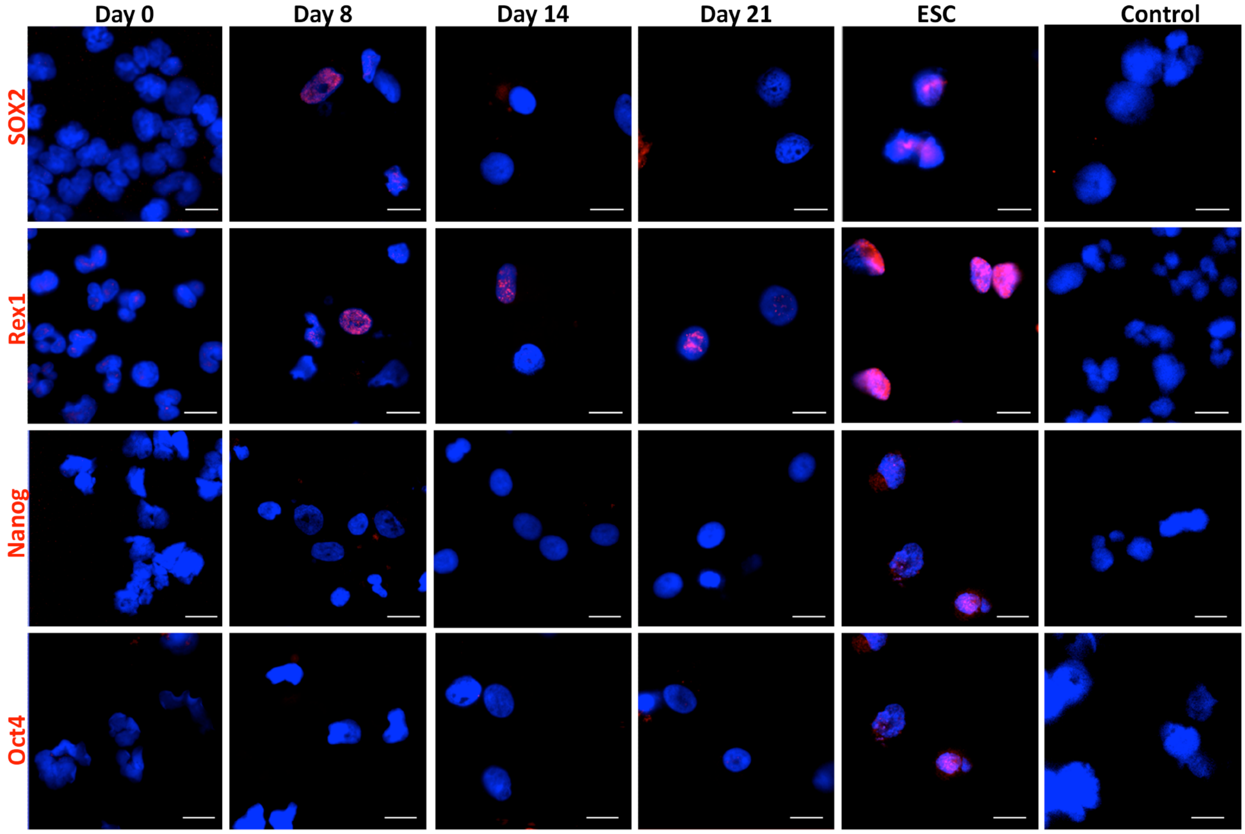

Supplementary Figure 11: Immunostaining of cultured cells at different time points (Days 0, 8, 14 and 21) for embryonic stem cell (ESC)-associated biomarkers (SOX2, Rex1, Nanog and Oct4). Cells were positive for some ESC biomarkers, namely Rex1. Boxed images (marked in white) provide examples of a distinct minority phenotype from the majority of cells. Negative controls for the antibodies are provided in the last column using blood cells from Day 0 lysed blood. Scale bar, 20 μm.

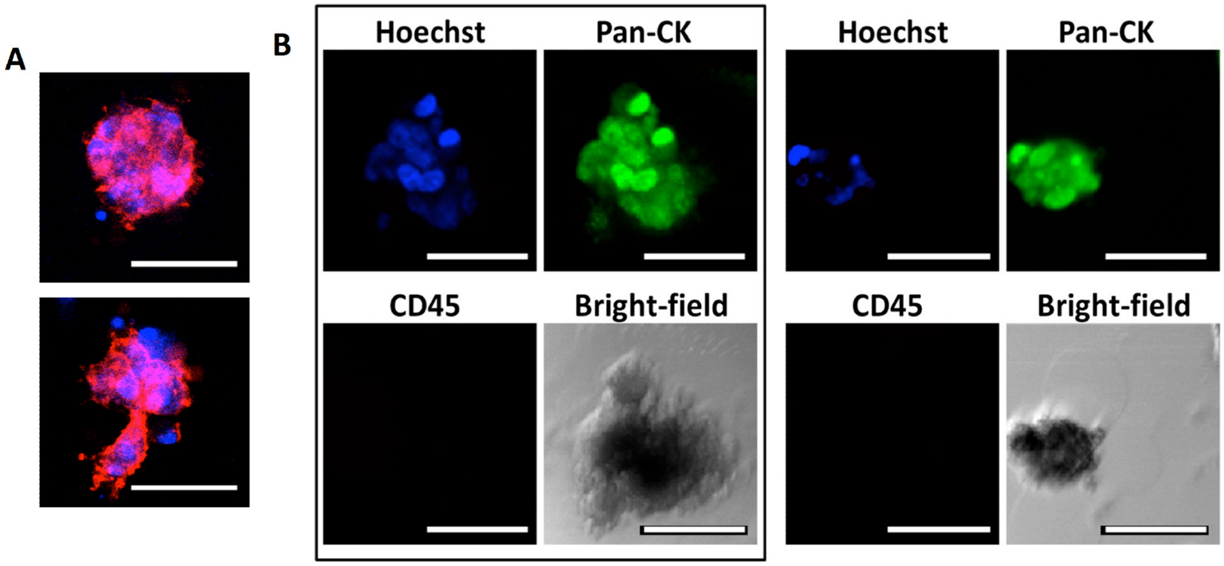

Supplementary Figure 12: Formation of spheroids from Day 14 cultures. A. Day 14 cultures can be transferred into 3D Geltrex® for further expansion into spheroids. *Left*: Spheroids stained for actin and Hoechst. *Right*: Some cells were observed to be migrating out of the spheroids into the surrounding matrix. Scale bar is 100 μm. **B.** Cultures can also be transferred into ultra-low adhesive dishes for propagation as spheroids over 10 days, and can withstand passages for at least 4 to 6 times. Resultant spheroids are stained for Hoechst, pan-CK and CD45 antibodies. Cells are mostly Hoechst+/pan-CK+/CD45-. Scale bar is 100 μm.

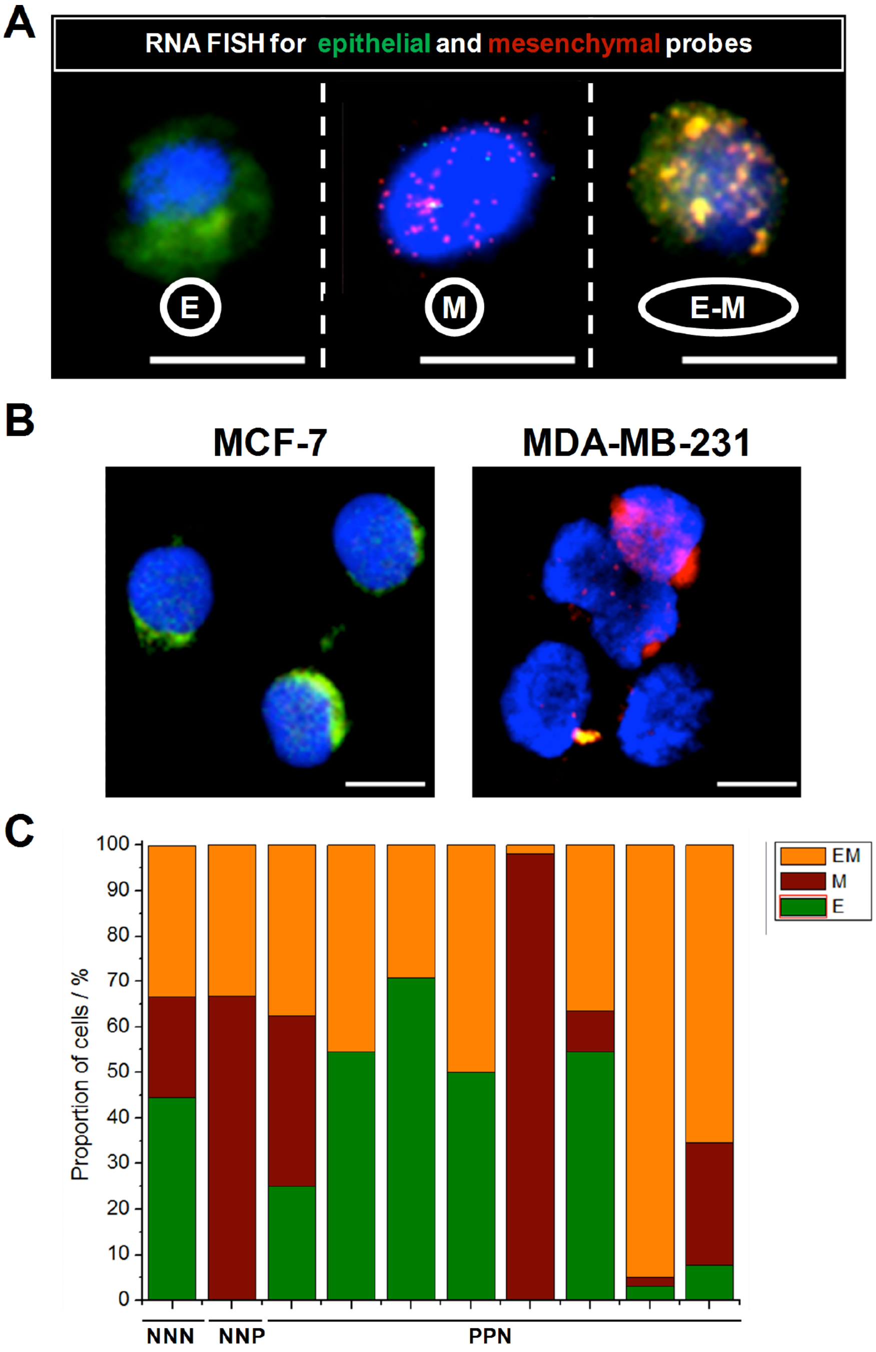

Supplementary Figure 13: EMT status of cultured cells. A. RNA FISH of Day 14 cultured cells with green (488)-labelled epithelial (CK7, CK8, CK18, CK19, CDH1, TFF1, FOXA1, AGR2 and GATA3) and red (550)-labelled mesenchymal (PTX3, SERPINE2, Vimentin, Fascin) gene probes. E, Epithelial; M, Mesenchymal; EM, Epithelial-Mesenchymal. Cells were considered as E if green:red signal ratio ≥ 2. Cells were classified as M if red: green signals ≥2. Scale bar, 20 μm. **B.** RNA FISH of the probes in (A) for control cell lines MCF-7 (epithelial) and MDA-MB-231 (mesenchymal). MCF-7 cells demonstrated mostly epithelial gene expression, whereas MDA-MB-231 was positive for mesenchymal gene probes. Scale bar, 20 μm. **C.** Proportion of cells from eight Day 14 cultures of 10 samples with E, M and EM status. PPN: Estrogen positive/progesterone positive/HER2 negative samples (PPN) (n = 8) contained a significant number of EM cells; one sample is fully M. NNN: Estrogen negative/progesterone negative/HER2 negative. NNP: Estrogen negative/progesterone negative/HER2 positive. Each bar corresponds to the respective sample as numbered (x-axis). The x-axis indicates the estrogen, progesterone and HER2 status of the patient.

Supplementary Table 1A: Clinicopathological characteristics of newly diagnosed breast cancer patients from P2A/P2B (doxorubicin/cyclophosphamide (AC) with or without Sunitinib) clinical trial, including time of blood withdrawal, demographics, tumor grade and phenotype, cancer stage, presence of metastasis, type of neoadjuvant therapy, treatment response and culture status. Samples that did not form multilayered clusters are indicated as N (no), whereas those that formed multilayered clusters are labelled as Y (yes). C, Chinese; M, Malay; I, Indian; O, Others; IDC, invasive ductal carcinoma; ILC, invasive lobular carcinoma; ER, estrogen; PR, progesterone; HER2, human epidermal growth factor receptor 2; For ER/PR/HER2, P, Positive; N, Negative. AC, doxorubicin/cyclophosphamide; Post-Sunitinib pre-AC is considered as ≤ 3 weeks after treatment; pre-surgery and post-surgery are considered as > 5 weeks after treatment. Shaded regions consist of samples used for analysis in Figure 5A.

Supplementary Table 1B: Clinicopathological characteristics of breast cancer patients with refractory metastatic disease under the CTB (refractory cancer) clinical trial, including time of blood withdrawal, demographics, tumor grade and phenotype, cancer stage, presence of metastasis, type of neoadjuvant therapy, treatment response and culture status. Samples that did not form multilayered cell cluster-like structures are indicated as N (no), whereas those that formed multilayered cell cluster-like structures are labelled as Y (yes). C, Chinese; M, Malay; I, Indian; O, Others; IDC, invasive ductal carcinoma; ILC, invasive lobular carcinoma; ER, estrogen receptor; PR, progesterone receptor; HER2, human epidermal growth factor receptor 2; AC, doxorubicin/ cyclophosphamide; FAC, 5-fluouracil/ doxorubicin/cyclophosphamide; P, Positive; N, Negative. Post-Sutent pre-AC are considered as ≤ 3 weeks after treatment; pre-surgery were considered as 3–5 weeks after treatment; post-surgery were considered as > 5 weeks after surgery.

Supplementary Table 1C: Clinicopathological characteristics of breast cancer patients under the PCL (mostly newly diagnosed, no metastasis) clinical trial, including time of blood withdrawal, demographics, tumor grade and phenotype, cancer stage, type of therapy, treatment response and culture status. Samples that did not form multilayered cell cluster-like structures are indicated as N (no), whereas those that formed multilayered cell cluster-like structures are labelled as Y (yes). C = Chinese; M, Malay; I, Indian; O, Others; IDC, invasive ductal carcinoma; ILC, invasive lobular carcinoma; ER, estrogen receptor; PR, progesterone receptor; HER2, human epidermal growth factor receptor 2; P, Positive; N, Negative. Post-Sutent pre-AC are considered as ≤ 3 weeks after treatment; pre-surgery were considered as 3–5 weeks after treatment; post-surgery were considered as > 5 weeks after surgery.

Supplementary Table 1D: Clinicopathological characteristics of breast cancer patients under the CES (early-stage cancer, no metastatic sites) clinical trial, including time of blood withdrawal, demographics, tumor grade and phenotype, cancer stage, type of therapy and culture status. Samples that did not form multilayered cell cluster-like structures are indicated as N (no), whereas those that formed multilayered cell cluster-like structures are labelled as Y (yes). C, Chinese; M, Malay; I, Indian; O, Others; IDC, invasive ductal carcinoma; ILC, invasive lobular carcinoma; ER, estrogen receptor; PR, progesterone receptor; HER2, human epidermal growth factor receptor 2; FAC, 5-fluouracil/ doxorubicin/cyclophosphamide; TCH, post-adjuvant Herceptin+Docetaxel+Carbo platin; FEC, Fluorouracil, Epirubicin, and Cyclophosphamide; P, Positive; N, Negative. Post-Sutent pre-AC are considered as ≤3 weeks after treatment; pre-surgery were considered as 3–5 weeks after treatment; post-surgery were considered as >5 weeks after surgery.

Supplementary Table 2: CK+ cell count/ml of blood

Sample no	CK+ cell count/ml of blood		Amplification(fold change)
	Day 0	Day 14	
1	220	6912	31.4
2	165	6451	39.0
3	154	1578	10.2
4	120	1901	15.8
5	104	4506	43.6
6	103	512	5.0
7	77	666	8.6
8	51	307	6.0
9	0	1741	NA
10	0	614	NA

Supplementary Table 3: Demographics for healthy volunteers

No	Race	Age	Multilayered cluster	With persistent blood cells
1	I	26	N	Y
2	C	31	N	Y
3	C	28	N	Y
4	C	24	N	N
5	C	25	N	Y
6	C	25	N	Y
7	C	40	N	Y
8	M	25	N	Y
9	C	36	N	Y
10	I	36	N	Y
11	C	30	N	N
12	O	67	N	Y
13	C	26	N	Y
14	C	27	N	Y
15	C	28	N	N
16	I	27	N	Y

C, Chinese; M, Malay; I, Indian; O, Others; N, no; Y, yes.

Supplementary Table 4: List of antibodies

**Stem cells**	**ESC**	Rex1	Abcam	IgG (Rabbit)	Mouse	1 to 250	Fig. S11
		SOX2	Abcam	IgG (Rabbit)	Mouse, Rat, Chicken, Human, Zebrafish, Quail, Rainbow Trout, Spotted Catshark (Scyliorhinus canicula), Thornback ray	1 to 250	
		OCT4	Abcam	IgG (Rabbit)	Mouse, Rat, Human, Pig, Macaque Monkey	1 to 250	
		NANOG	Abcam	IgG (Rabbit)	Mouse, Human, Monkey	1 to 250	
	**CSC**	CD44	MACs	IgG1 (Rat)	Rhesus monkey (Macaca mulatta), cynomolgus monkey (Macaca fascicularis), chimpanzee (Pan troglodytes), baboon, human, pig, cow, horse, cat, dog	1 to 100	Fig. S8, S9
		CD24	MACs	IgG1 (Rat)	Rhesus monkey (Macaca mulatta), cynomolgus monkey (Macaca fascicularis), chimpanzee (Pan troglodytes), baboon, human, pig, cow, horse, cat, dog	1 to 100	
	**MSC**	CD90	Abcam	IgG (Rabbit)	Rat, Human	1 to 250	Fig. S7
	**HSC**	CD34	BD	IgG_{2a}, κ (Rat)	Mouse	1 to 250	
**MSC derivatives**	**Adipocyte**	FABP4	Abcam	IgG (Rabbit)	Mouse, Human	1 to 250	
	**Chondrocyte**	Aggrecan	Abcam	IgG_1 (Mouse)	Cow, Human	1 to 250	
	**Osteocyte**	Osteocalcin	Abcam	IgG (Rabbit)	Human	1 to 250	
	**Cardiomyocytes**	Troponin T	Abcam	IgG2b (Mouse)	Mouse, Rat, Rabbit, Goat, Cow, Cat, Dog, Human, Pig, Fish	1 to 250	

(*Continued*)

Stem cells	ESC	Rex1	Abcam	IgG (Rabbit)	Mouse	1 to 250	Fig. S9
**WBC**	**Leukocytes**	CD45	MACs	IgG2a (Mouse)	Human	1 to 100	Fig. 2, S4, S8, S9
		CD18	Abcam	IgG (Rabbit)	Human	1 to 250	
	**Macrophages**	CD68	Abcam	IgG_1 (Mouse)	Mouse, Rat, Human	1 to 250	
		MIF	Abcam	IgG (Rabbit)	Mouse, Rat	1 to 250	Fig. 2
	**Monocytes**	CD14	Abcam	IgG (Goat)	Human	1 to 250	
		CD16	Abcam	IgM (Mouse)	Human	1 to 250	
	**Megakaryocytes**	Thrombospondin-1	Abcam	IgM (Mouse)	Cow, Human	1 to 250	
	**Natural killer cells**	CD56	Abcam	IgG (Rabbit)	Human	1 to 250	
**Endothelial cell**		CD31	BD	IgG_1 (Mouse)	Mouse	1 to 250	
		VWF	Abcam	IgG (Rabbit)	Rat, Guinea pig, Cow, Dog, Human, Pig	1 to 250	
**Epithelial cell**		E-cadherin	Abcam	IgG1 (Rat)	Mouse, Dog, Human	1 to 250	Fig. 3
		Pan-cytokeratin	MACs	IgG1 (Rat)	Rhesus monkey (Macaca mulatta), cynomolgus monkey (Macaca fascicularis), chimpanzee (Pan troglodytes), baboon, human, pig, cow, horse, cat, dog	1 to 100	Fig. 2, S4, S9, S12
		Cytokeratin 5	Abcam	IgG (Rabbit)	Mouse, Human	1 to 250	Fig. 3
		Cytokeratin 7	Abcam	IgG_1 (Mouse)	Human	1 to 250	
		Cytokeratin 8	Dako	IgG_1 (Mouse)	Human	1 to 250	
		Cytokeratin 18	Dako	IgG_1 (Mouse)	Human	1 to 250	
		Cytokeratin 19	Dako	IgG_1 (Mouse)	Human	1 to 250	
		EpCAM	MACs	IgG_1 (Mouse)	Human	1 to 100	
**Mesenchymal cell**		Vimentin	Sigma-Aldrich	IgG_1 (Mouse)	human, pig, chicken, or rat	1 to 250	Fig. 3, S4
		Fascin	Abcam	IgG_1 (Mouse)	Human, Pig, Gerbil, Sea Urchin	1 to 250	Fig. 3
**Others**		Ki67	Abcam	IgG (Rabbit)	Mouse, Rat, Sheep, Rabbit, Horse, Cow, Dog, Human, Pig, Indian Muntjac, Monkey, Chinese Hamster, Marmoset (common), Syrian Hamster	1 to 250	Fig. S6

Supplementary Table 5: List of probes for in-situ hybridization

Specificity	Probes	Company	Chromophore	Reference
**Epithelial cells**	E-cadherin	iDNA	Green	Fig. S13
	TFF1	iDNA	Green	
	FOXA1	iDNA	Green	
	AGR2	iDNA	Green	
	GATA3	iDNA	Green	
	Cytokeratin 7	iDNA	Green	
	Cytokeratin 8	iDNA	Green	
	Cytokeratin 18	iDNA	Green	
	Cytokeratin 19	iDNA	Green	
**Mesenchymal cells**	Fascin	iDNA	Red	
	Vimentin	iDNA	Red	
	PTX3	iDNA	Red	
	SERPIN2	iDNA	Red	
**Breast cancer cells**	CCND1	Empire Genomics	Red	Figure 4
	HER2	Kreatech	Red	
	FGFR1	Empire Genomics	Red	
	MYC	Empire Genomics	Red	
	TOP2A	Abbott	Red	
	ZNF217	Abbott	Red	
	CEN17	Kreatech	Green	

Supplementary Table S6: List of 14 post-treatment samples from patients with refractory cancer computed for survival statistics in Figure 5B

Sample	ID	Survived	With early radiological progressive disease (within 3 months)	OS (months)	Cluster
1	CTB 002	Y	Y	28.41	N
2	CTB 004	N	Y	13.98	N
3	CTB 005	N	Y	8.81	Y
4	CTB 006	N	N	5.72	Y
5	CTB 007	N	Y	4.14	Y
6	CTB 009	N	Y	6.12	Y
7	CTB 012	N	Y	8.94	N
8	CTB 014	N	NA	16.54	Y
9	CTB 015	N	Radiological response after 2 months	6.35	N
10	CTB 016	Y	N	16.97	N
11	CTB 017	Y	N	21.21	N
12	CTB 019	N	N	14.21	Y
13	CTB 026	Y	N	8.22	Y
14	CTB 029	Y	NA	5.56	Y

OS, overall survival; Y, yes; N, no; NA, not applicable.
